# A generalized theory of separable effects in competing event settings

**DOI:** 10.1007/s10985-021-09530-8

**Published:** 2021-09-01

**Authors:** Mats J. Stensrud, Miguel A. Hernán, Eric J Tchetgen Tchetgen, James M. Robins, Vanessa Didelez, Jessica G. Young

**Affiliations:** 1 Department of Mathematics, Ecole Polytechnique Fédérale de Lausanne, Lausanne, Switzerland; 2 Department of Epidemiology, Harvard T. H. Chan School of Public Health, Boston, USA; 3 Department of Biostatistics, Harvard T. H. Chan School of Public Health, Boston, USA; 4 CAUSALab, Harvard T.H. Chan School of Public Health, Boston, USA; 5 Department of Statistics, The Wharton School, University of Pennsylvania, Philadelphia, USA; 6 Leibniz Institute for Prevention Research and Epidemiology - BIPS, Bremen, Germany; 7 Faculty of Mathematics/Computer Science, University of Bremen, Bremen, Germany; 8 Department of Population Medicine, Harvard Medical School and Harvard Pilgrim Health Care Institute, Boston, USA

**Keywords:** Causal inference, Competing events, Effect decomposition, G-formula, Hazard functions, Separable effects

## Abstract

In competing event settings, a counterfactual contrast of cause-specific cumulative incidences quantifies the total causal effect of a treatment on the event of interest. However, effects of treatment on the competing event may indirectly contribute to this total effect, complicating its interpretation. We previously proposed the *separable effects* to define direct and indirect effects of the treatment on the event of interest. This definition was given in a simple setting, where the treatment was decomposed into two components acting along two separate causal pathways. Here we generalize the notion of separable effects, allowing for interpretation, identification and estimation in a wide variety of settings. We propose and discuss a definition of separable effects that is applicable to general time-varying structures, where the separable effects can still be meaningfully interpreted as effects of modified treatments, even when they cannot be regarded as direct and indirect effects. For these settings we derive weaker conditions for identification of separable effects in studies where decomposed, or otherwise modified, treatments are not yet available; in particular, these conditions allow for time-varying common causes of the event of interest, the competing events and loss to follow-up. We also propose semi-parametric weighted estimators that are straightforward to implement. We stress that unlike previous definitions of direct and indirect effects, the separable effects can be subject to empirical scrutiny in future studies.

**Supplementary Information:**

The online version supplementary material available at 10.1007/s10985-021-09530-8.

## Introduction

Researchers are often interested in treatment effects on an event of interest that is subject to competing events, that is, events that make it impossible for the event of interest to subsequently occur. For example, when the event of interest is kidney injury, death is a competing event because any individual who dies prior to kidney injury cannot subsequently suffer from kidney injury. Several estimands have already been suggested for causal inference in competing events settings with known shortcomings.

A counterfactual contrast in cause-specific cumulative incidences (risks) quantifies the *total effect* of the treatment on the event of interest through all causal pathways. Here we intentionally use the term *total effect* to bridge competing event settings to results from mediation analysis (Stensrud et al. 2020; Robins and Richardson 2010; Robins et al. 2020). When the treatment affects competing events, the total effect also partly includes pathways mediated by these competing events (Robins 1986; Young et al. 2020). For example, a harmful total effect of blood pressure therapy on the risk of kidney injury may be due to a biological side-effect on the kidneys, but could also be fully or partly explained by a protective treatment effect on cardiovascular death. As previously discussed (Robins 1986; Young et al. 2020; Tchetgen Tchetgen 2014), other popular estimands in competing events settings do not resolve this interpretational problem. This includes popular approaches based on cause-specific or subdistribution hazard models, even if formulated in terms of counterfactuals. Hazard based contrasts are broadly problematic as causal contrasts (Martinussen et al. 2020; Robins 1986; Hernán 2010; Stensrud and Hernán 2020), also in competing event settings (Young et al. 2020).

Other estimands that have been considered for causal inference in the face of competing events that do have a causal interpretation include the controlled direct effects (Robins and Greenland 1992; Young et al. 2020) and pure (natural) effects (Robins and Greenland 1992; Pearl 2009). However, these estimands refer to treatment effects under unspecified interventions on the competing events; in the example on blood pressure therapy, we would need to consider an intervention that “eliminates” death from all causes. Such hypothetical interventions are irrelevant in nearly every practical setting. Furthermore, identification of pure (natural) effects relies on counterfactual assumptions across different “worlds” that are untestable, even in principle (Robins and Richardson 2010).

To address these problems, we recently proposed the *separable effects* for causal inference in competing event settings (Stensrud et al. 2020), inspired by Robins and Richardson’s extended graphical approach to mediation analysis (Robins and Richardson 2010; Didelez 2018; Robins et al. 2020). Given a plausible decomposition of the treatment into different components, we defined these effects as counterfactual contrasts indexed by hypothetical interventions that assign these components different values. The separable effects have clear advantages over existing causal estimands, explicitly quantifying the effects of modified treatments and forcing investigators to sharpen specifications about their causal question of interest, in turn, fostering new ideas and hypotheses about future real-world treatment strategies (Robins and Richardson 2010; Stensrud et al. 2020). The separable effects generally rely on weaker assumptions for identification than the alternative estimands outlined above (Robins and Richardson 2010; Stensrud et al. 2020; Didelez 2018). They do not conceptualize hypothetical interventions that eliminate competing events and avoid cross-world assumptions, which can never be subject to empirical scrutiny. Instead, the separable effects can, at least in principle, be directly identified in a future experiment where the treatment components are assigned different values. However, the interpretation and identification of separable effects given in our initial work (Stensrud et al. 2020) relied fundamentally on the assumption that there exist only pre-treatment common causes of the competing event and event of interest. This assumption, which has implications for both the interpretation and identification of the separable effects, is overly restrictive in many real-world applications thus limiting the applicability of these initial results.

Here, we generalize the early results of Stensrud et al. (2020) to allow more realistic data structures, such that time-varying covariates and common causes of the competing event and event of interest can exist. Our results substantially broaden the theory of separable effects, providing an explicit and transparent approach to reasoning around mechanism in general competing events settings and, in turn, translating this reasoning into a statistical analysis. Specifically, in this paper we formalize conditions that allow particular mechanistic interpretations of separable effects in a range of settings. The strongest of these conditions ensures that the separable effects can be interpreted as the *direct effects* of the treatment on the event of interest (capturing *all* treatment effects on the event of interest *not via* treatment effects on competing events) and the *indirect effects* of the treatment on the event of interest (capturing *all* treatment effects on the event of interest *only via* treatment effects on competing events). However, we show that weaker conditions also allow practically relevant mechanistic interpretations of these effects – e.g. capturing *some* (but not all) direct effects; that is, *some* (but not all) treatment effects on the event of interest not via effects on competing events. We formalize conditions for identification of the separable effects in this general setting where baseline and time-varying covariates are measured. Interestingly, the identification formulas are actually identical to formulas contained in Shpitser (2013), although these identification results have different interpretations and require different assumptions. Finally we present semi-parametric weighted estimators of the separable effects under this time-varying data structure.

The manuscript is organized as follows. In Sect. 2, we describe the observed data structure in which the event of interest is subject to competing events and both baseline and time-varying covariates are measured. In Sect. 3, we review the definition of the total effect on an event of interest subject to competing events. In Sect. 4, we define a generalized decomposition assumption that is agnostic to the mechanism by which the treatment exerts effects on the competing event and the event of interest. We also formally define the separable effects. In Sect. 5, we formalize a range of conditions by which the treatment components may exert effects on future outcomes and explain the interpretation of the separable effects in each case. In Sect. 6, we give conditions that allow identification of the separable effects under the observed data structure by a particular g-formula (Robins 1986). We also generalize identification results to allow for censored data. In Sect. 7, we provide two weighted representations of the g-formula for the separable effects and use these representations to motivate weighted estimators, which are supplemented with sensitivity analysis techniques. We also apply these results to a randomized study of the effect of intensive versus standard blood pressure therapy on acute kidney injury. In Sect. 8, we provide a discussion.

## Observed data structure

We consider an experiment in which $i=1,\ldots ,n$ individuals are randomly assigned to one of two treatment arms $A\in \{0,1\}$ at baseline (e.g. $A=0$ and $A=1$ denote assignment to standard and intensive blood pressure therapy, respectively). We assume that observations are independent and identically distributed and suppress the *i* subscript. Let $k=0,1,2,...,K+1$ be equally spaced time intervals with interval $k=0$ corresponding to baseline (the interval of randomization) and interval $k=K+1$ the maximum follow-up of interest at or before the administrative end of follow-up (e.g. 60 months).

For $k>0$, let $Y_{k}$ and $D_{k}$ denote indicators of an event of interest (e.g. kidney injury) and a competing event (e.g. death) by interval *k*, respectively, and $L_{k}$ a vector of individual time-varying covariates in that interval. Define $D_{0} \equiv Y_{0} \equiv 0$, i.e. the population is restricted to those alive and at risk of all events prior to randomization. Further, define $L_0$ as a vector of pre-randomization covariates. We denote the history of a random variable by an overbar, e.g. $\bar{Y}_{k}=(Y_{0},Y_{1},...,Y_{k})$ is the history of the event of interest through interval *k*, and the future of a random variable through $K+1$ by an underline, e.g. $\underline{Y}_{k}=(Y_{k},Y_{k+1},...,Y_{K+1})$. Throughout, we assume a temporal order $(D_{k},Y_{k},L_{k})$ in each interval $k > 0$. As interval lengths become arbitrarily small, this temporal order assumption is guaranteed because the probability that two events of any type occur within that interval approaches zero (equivalent to the common assumption in survival analysis of no tied event times). When the event of interest is terminal (e.g. death due to prostate cancer), the time-varying event history $\overline{D}_{K+1},\overline{Y}_{K+1}$ coincides with the more familiar “competing risks” data structure $\{\tilde{T}=\min (T,G),J\}$ for *T* the time to failure from any cause, *G* a censoring time and *J* an indicator of cause of failure such that $J=0$ when $\tilde{T}=G$ and $J>0$ otherwise (e.g. $J=1$ if failure from the event of interest and $J=2$ if failure from the competing event). Regardless of whether the event of interest is terminal (we have a “competing risks” data structure) or nonterminal (we have a “semicompeting risks” data structure), defining the observed data structure in terms of time-varying failure status, as opposed to summarized failure times, is essential for understanding identification and interpretation of many causal estimands in survival analysis, including those considered here. Further, it avoids the assumption that there exists a censoring time *G* for individuals who are observed to fail (e.g. die) during the follow-up.^1^ Importantly, our results throughout apply regardless of whether the event of interest is terminal or nonterminal.

By definition of a competing event, if an individual experiences this event by interval *k* without history of the event of interest $(Y_{k-1}=0, D_k=1)$ then $\underline{Y}_{k}=0$; an individual who experiences the competing event cannot subsequently experience the event of interest, regardless of whether this is terminal or nonterminal, that is, regardless of whether it is also the case that $Y_{k-1}=1$ determines ${\textit{D}}_{k}=0$. For ease of presentation, we will assume no individual is censored by loss to follow-up (that is, $\overline{D}_{K+1},\overline{Y}_{K+1}$ is fully observed for all individuals randomized at baseline) until Sect. 6.3.

## The total treatment effect on the event of interest

For any individual in the study population and for $k \in \{0,\ldots ,K\}$, let $Y_{k+1}^{a}$ be the indicator of the event of interest by interval $k+1$ had, possibly contrary to fact, he/she been assigned to $A=a$. The contrast

1 $$\begin{aligned} \Pr (Y_{k+1}^{a=1}=1) \text { vs. } \Pr (Y_{k+1}^{a=0}=1) \end{aligned}$$

is then the cause-specific cumulative incidence function, which we intentionally denote a *total effect* of treatment *A* on the risk of the event of interest by interval $k+1$ in this study population. This effect includes treatment effects on the competing event (Young et al. 2020).

**Fig. 1:**
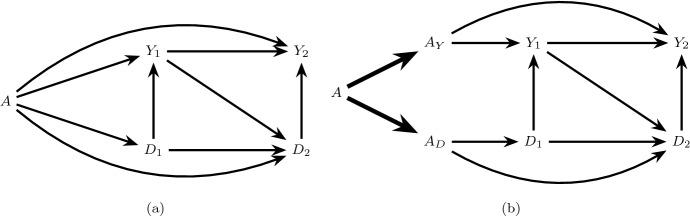
The directed acyclic graph (DAG) in **a** represents a restrictive data generating assumption on the observed data structure such that there are no common causes of the event of interest and the competing event at any time. The extended DAG in **b** is an augmented version of the graph in **a** representing a treatment decomposition satisfying the generalized decomposition assumption. The bold arrows encode deterministic relationships

We will use causal directed acyclic graphs (DAGs) (Pearl 2009) to represent underlying assumptions on the mechanisms by which random variables in the study of Sect. 2 are generated. A causal DAG must represent all common causes of any variable represented on the DAG. For example, the causal DAG in Fig. 1a represents a generally restrictive assumption on this data generating process for a subset of time points because it depicts no common causes (measured or unmeasured) of event status over time. Throughout we will assume that causal DAGs represent a Finest Fully Randomized Causally Interpreted Structural Tree Graph (FFRCISTG) model, a type of counterfactual causal model that includes the non-parametric structural equation model with independent errors (NPSEM-IE) (Robins 1986; Robins et al. 2020; Robins and Richardson 2010; Pearl 2009; Shpitser et al. 2020) as a submodel, and we assume that statistical independencies in the data are faithful to the DAG (Verma and Pearl 1991).

The total effect of *A* on $Y_2$ in Fig. 1a includes all directed (causal) paths between *A* and $Y_2$. This includes causal paths that do not capture the treatment’s effect on the competing event (e.g.  $A \rightarrow Y_1 \rightarrow Y_2$ and $A \rightarrow Y_2$) as well as causal paths that capture this effect (e.g. $A \rightarrow D_1 \rightarrow D_2 \rightarrow Y_2$ and $A \rightarrow D_2 \rightarrow Y_2$). While the total effect can be straightforward to identify from a study in which *A* is randomly assigned, its interpretation is complicated when pathways like $A\rightarrow D_2\rightarrow Y_2$ in Fig. 1a are present (Young et al. 2020; Stensrud et al. 2020). For example, a harmful total effect of intensive versus standard blood pressure therapy on kidney injury, i.e. $\Pr (Y_{k+1}^{a=1}=1)>\Pr (Y_{k+1}^{a=0}=1)$, may be wholly or partially explained by one of these pathways (e.g. a protective effect of intensive therapy on death).

## Generalized decomposition assumption and separable effects

Consider the following assumption:

- Generalized decomposition assumption
  2 $$\begin{aligned}&\text {The treatment }A\hbox { can be decomposed into two binary} \nonumber \\&\text {components }A_Y\in \{0,1\}\hbox { and }A_D\in \{0,1\}\hbox { such that,} \nonumber \\&\text {in the observed data, the determinism} \nonumber \\&A\equiv A_D\equiv A_Y\, \text {holds, } \text {but in a future study},\,A_Y\hbox { and }A_D \nonumber \\&\text {could, in principle, be assigned different values.} \end{aligned}$$
  Let $$\overline{Z}_k$$, $$k\in \{0, \dots ,K\}$$, be the vector of all (direct or indirect) causes of $$ {Y}_{k+1}$$ and/or $$ {D}_{k+1}$$, excluding $$(A_Y,A_D)$$, and $$Z_j, j=0,\dots ,k$$, are these causes in interval *j*, where *V* is a cause of *W* if changing the value of *V* may result in a change in the value of *W*. We intentionally distinguish time-varying covariates that are measured in our study, $$\overline{L}_k$$, from $$\overline{Z}_k$$; $$\overline{L}_k$$ could e.g. be a subset of $$\overline{Z}_k$$. We shall see that the variables in $$\overline{Z}_k$$ are needed to express the substantive meaning of particular separable effects. We will need to make assumptions about the nature of $$\overline{L}_k$$ to reason about whether separable effects can be identified using only what was measured in our study, which will require that $$\overline{L}_k$$ is a subset of $$\overline{Z}_k$$. There is not surprisingly a link between these interpretation and identification tasks as we formalize in Sect. 6.1. We keep these tasks separate because explicit reasoning about interpretation of separable effects provides value for the design of future studies even if identification in the current study fails given limitations of measurement. This may be the case if causal reasoning about questions and assumptions occurs after the data collection is complete.
  We also assume that an intervention that assigns $A=a$ results in the same outcome as an intervention that assigns $A_Y=A_D=a$, that is,
  3 $$\begin{aligned}&Y_{k+1}^{a_Y=a,a_D=a}=Y_{k+1}^{a}, \nonumber \\&D_{k+1}^{a_Y=a,a_D=a}=D_{k+1}^{a}, \nonumber \\&Z_{k+1}^{a_Y=a,a_D=a}=Z_{k+1}^{a}, \quad k \in \{0, \dots ,K\}, \end{aligned}$$
  where $W_{k+1}^{a_Y,a_D}$ for $W_{k+1} \in \{Y_{k+1},D_{k+1},Z_{k+1}\}$ $k \in \{0,\ldots ,K\}$, is the value of $W_{k+1}$ had, contrary to fact, he/she been assigned the components $A_Y=a_Y$ and $A_D=a_D$, in place of assignment to a value of the original treatment *A*.

Beyond (3), the generalized decomposition assumption makes no mechanistic assumptions on the effects exerted by $A_Y$ and $A_D$. We will consider different examples of treatment decompositions in Sect. 5 where, unlike those considered by Stensrud et al. (2020), the effects exerted by $A_Y$ and $A_D$ are not necessarily direct and indirect effects. Furthermore, in Appendix A we consider straightforward further generalizations of our results to settings where $A_Y$ and $A_D$ are not a decomposition of *A*, violating (2), but are still treatments satisfying (3).

For $k \in \{0,\ldots ,K\}$, the contrast

4 $$\begin{aligned} \Pr (Y_{k+1}^{a_Y=1,a_D}=1)\text { vs. }\Pr (Y_{k+1}^{a_Y=0,a_D}=1), \quad a_D \in \{0,1\}, \end{aligned}$$

quantifies the causal effect of the $A_Y$ component on the risk of the event of interest by $k+1$ under an intervention that assigns $A_D=a_D$ (Stensrud et al. 2020; Robins and Richardson 2010; Robins et al. 2020). Similarly

5 $$\begin{aligned} \Pr (Y_{k+1}^{a_Y,a_D=1}=1)\text { vs. }\Pr (Y_{k+1}^{a_Y,a_D=0}=1), \quad a_Y \in \{0,1\}, \end{aligned}$$

quantifies the causal effect of the $A_D$ component on the risk of the event of interest by $k+1$ under an intervention that assigns $A_Y=a_Y$.

We will refer to (4) as the $A_Y$ *separable effect under* $A_D=a_D$, $a_D\in \{0,1\}$ and (5) as the $A_D$ *separable effect under* $A_Y=a_Y$, $a_Y\in \{0,1\}$. Given the generalized decomposition assumption, the total effect can be expressed as a sum of particular $A_Y$ and $A_D$ separable effects, for example,

$$\begin{aligned}&\Pr (Y_{k+1}^{a_Y=1,a_D=1}=1)-\Pr (Y_{k+1}^{a_Y=0,a_D=1}=1) \\&\quad +\Pr (Y_{k+1}^{a_Y=0,a_D=1}=1)-\Pr (Y_{k+1}^{a_Y=0,a_D=0}=1) \\&\quad = \Pr (Y_{k+1}^{a=1}=1)-\Pr (Y_{k+1}^{a=0}=1). \end{aligned}$$

## Isolation conditions and interpretation of separable effects

In this section, we consider conditions, beyond the generalized decomposition assumption, under which we can ascribe a more precise interpretation to the separable effects (4) and (5). The strongest of these assumptions allows interpretation of these effects as the separable direct and indirect effects of Stensrud et al. (2020).

To formally define these additional conditions, we will first review the definition of an *extended causal DAG* (Robins and Richardson 2010): an extended causal DAG augments the original causal DAG with additional nodes representing components of the treatment, and bold edges representing the deterministic relation between these components and the full treatment in the observed data. For example, the extended causal DAG in Fig. 1b is an augmented version of the causal DAG in Fig. 1a, which generalizes the extended DAG in Figure 3 of Robins and Richardson (2010) to time-dependent mediators and outcomes. The extended causal DAG also encodes assumptions, not represented on the original causal DAG, on the mechanisms by which each treatment component exerts effects on future variables. Arrows from $D_k$ to $Y_{k+j}$, $j>0$ (for example $D_1 \rightarrow Y_2$ in Fig. 1a, b) are unnecessary in our case, where time-varying mediators constitute competing events, but these arrows could have been included without changing any of our results.

### Full isolation

Consider an extended causal DAG in which *A* is decomposed into two components $A_Y$ and $A_D$ satisfying the generalized decomposition assumption, and define the following conditions:

6 $$\begin{aligned}&\text {The only causal paths from } A_Y \text { to } D_{k+1}, k=0,...,K \text { are directed} \nonumber \\&\text {paths intersected by } Y_{j}, j=0,...,k. \end{aligned}$$

7 $$\begin{aligned}&\text {The only causal paths from } A_D \text { to } Y_{k+1}, k=0,...,K \text { are directed} \nonumber \\&\text {paths intersected by } D_{j+1}, j=0,...,k. \end{aligned}$$

**Fig. 2:**
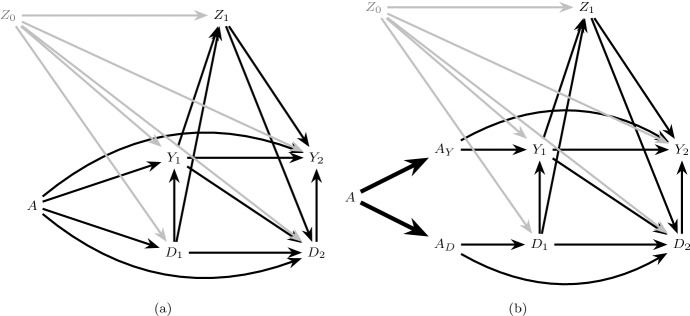
The causal DAG in **a** allows a pre-randomization common cause ($Z_0$) of $\underline{Y}_1$ and $\underline{D}_1$ and post-randomization common cause ($Z_1$) of $Y_2$ and $D_2$ but assumes $Z_1$ is not affected by treatment *A*. **b** is an extension of **a** satisfying full isolation

When both conditions (6) and (7) hold we will say there is *full isolation*. This assumption is satisfied in Fig. 1b which assumes there are no common causes of the event of interest and the competing event. It is also satisfied in Fig. 2b which allows the presence of both pre-randomization ($Z_0$) and post-randomization ($Z_1$) common causes.

Under the generalized decomposition assumption and full isolation, (4) are the separable direct effects of *A* on the risk of the event of interest by $k+1$, which do not capture the treatment’s effect on the competing event: that is, a distinct causal mechanism by which *A* directly affects the event of interest outside of *A*’s indirect effects through the competing event. Similarly, (5) are the separable indirect effects of *A* on this risk, which only capture the treatment’s effect on the competing event. Full isolation coincides with the settings considered by Stensrud et al. (2020), which allowed for the presence of pre-randomization, but not post-randomization, common causes of the event of interest and the competing event.

Returning to our running example, assume that the blood pressure treatment *A* can be decomposed into a component $A_Y$ that binds to receptors in the kidneys, e.g. by relaxing the efferent arterioles which is a well-known biological effect of commonly used blood pressure drugs such as angiotensin-converting-enzyme inhibitors (ACE) and angiotensin II receptor blockers (ARB), and a component $A_D$ that includes the remaining components of the antihypertensive therapy, some of which lead, for example, to reductions in systemic blood pressure.

Then, $A_Y=0$ and $A_Y=1$ are the levels (e.g., doses) of the $A_Y$ component under standard and intensive therapy, respectively, and $A_D=1$ and $A_D=0$ are defined analogously.

Full isolation would be satisfied in this case if (i) the $A_Y$ component only exerts effects on death through its effects on kidney function and (ii) the remaining $A_D$ component only exert effects on kidney function through its effects on survival. In Sect. 5.2, however, we argue that the assumption of full isolation may not be reasonable in this example.

### $A_Y$ partial isolation

**Fig. 3:**
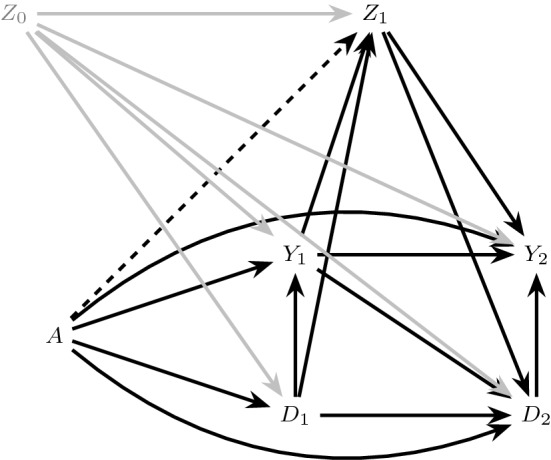
A causal DAG representing the assumption that $Z_1$, a common cause of $Y_2$ and $D_2$, may be affected by treatment *A* (dashed arrow)

The causal graphs in Figs. 1 and 2 make the restrictive assumption that there are no common causes of the event of interest and competing event that are, themselves, affected by treatment. In our running example, this assumption likely fails: a reduction in blood pressure may increase the risk of kidney injury (the event of interest) due to hypoperfusion of the kidneys (for example, when patients are dehydrated) (Aalen et al. 2019) and also may affect the risk of mortality (the competing event). Further, blood pressure itself clearly may be affected by the blood pressure treatment. The causal DAG in Fig. 3 depicts the more realistic assumption that blood pressure ($Z_1$) is both a possible common cause of future kidney injury $Y_2$ and mortality $D_2$ and also, itself, affected by treatment *A* (represented by the dashed arrow connecting *A* to $Z_1$).

**Fig. 4:**
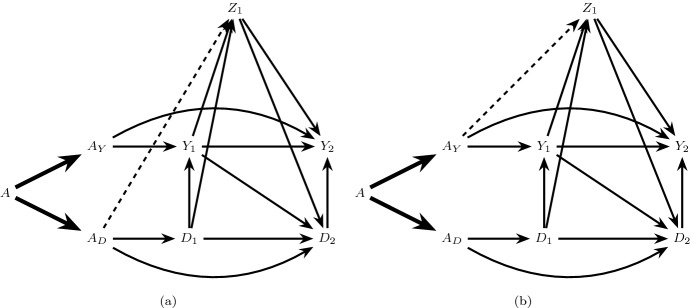
Extensions of the causal DAG in Fig. 3 illustrating partial isolation. The dashed arrow in **a** represents the $A \rightarrow Z_1$ relation in Fig. 3 under $A_Y$ partial isolation, and the dashed arrow in **b** represents $A_D$ partial isolation

Suppose, however, that the $A_Y$ component of the treatment *A* (that which binds to receptors in the kidneys) has no effect on blood pressure outside of its possible effect on kidney function, such that only the remaining components of treatment, $A_D$, can directly affect blood pressure. The extended DAG in Fig. 4a, which is one possible extension of the causal DAG in Fig. 3, represents this assumption by the dashed arrow from $A_D$ into $Z_1$ and the absence of an arrow from $A_Y$ into $Z_1$. In this case, condition (6) holds but (7) does not. When only the condition (6) holds, but (7) fails, we will say there is $A_Y$ *partial isolation*.

Unlike under full isolation, under $A_Y$ partial isolation, the $A_D$ separable effects (5) quantify *both* direct effects of the treatment on the event of interest not through the competing event (e.g. the path $A_D \rightarrow Z_1 \rightarrow Y_2$ in Fig. 4a) and indirect effects through the competing event (e.g. the path $A_D \rightarrow D_1 \rightarrow Y_1 \rightarrow Y_2$ in Fig. 4a).^2^ By contrast, the $A_Y$ separable effects *only* quantify direct effects not through the competing event. However, the $A_Y$ separable effects do not capture all direct effects in this case, because some of these pathways may originate from $A_D$ as described above. In the current example, the $A_Y$ separable effect evaluated at $a_D=1$ may be of particular clinical interest, quantifying the effect of assignment to the current intensive therapy containing all components versus a modified intensive therapy that lacks the component possibly affecting the kidneys.

### $A_D$ partial isolation

When (7) holds, but (6) fails, we will say there is $A_D$ *partial isolation*. $A_D$ partial isolation is represented in Fig. 4b, depicting an alternative augmentation of the causal DAG in Fig. 3. Under $A_D$ partial isolation, the $A_Y$ separable effects (4) quantify both direct effects of the treatment on the event of interest not through the competing event (e.g. the path $A_Y \rightarrow Z_1 \rightarrow Y_2$ in Fig. 4b) and indirect effects through the competing event (e.g. the path $A_Y \rightarrow Z_1 \rightarrow D_2 \rightarrow Y_2$ in Fig. 4b). By contrast, the $A_D$ separable effects *only* quantify indirect effects through the competing event. However, the $A_D$ separable effects do not capture all indirect effects in this case, because some of these pathways may originate from $A_Y$ as above.

As an example of $A_D$ partial isolation, trials have reported an increase in the risk of new-onset type 2 diabetes among patients assigned to statins (Sattar et al. 2010; Ridker et al. 2012). However, statins also reduce the risk of all-cause mortality, a competing event for type 2 diabetes onset (the event of interest). It is therefore unclear whether a total effect of statin treatment on type 2 diabetes is due a protective treatment effect on mortality, a biologically harmful process leading to type 2 diabetes onset or some combination.

Figure 3 illustrates a possible underlying causal structure for a trial with random assignment to statin therapy relating treatment assignment *A*, mortality $D_k$ and new-onset type 2 diabetes $Y_k$, $k=1,2$. Body weight ($Z_1$) is a possible common cause of both mortality and onset of type 2 diabetes which may also be affected by statin treatment. Consider a decomposition of *A* (represented in Fig. 4b) where $A_D$ may lead to increased risk of diabetes only by reducing mortality risk (e.g. through $A_D \rightarrow D_1 \rightarrow D_2 \rightarrow Y_2$, where the reduction in mortality risk is likely due to reduced levels of low density lipoprotein in the blood), while a second component $A_Y$ exerts unintended effects of statins on diabetes through body weight (e.g. through $A_Y \rightarrow Z_1 \rightarrow Y_2$). As in the previous example of blood pressure therapy and kidney injury, the $A_Y$ separable effect of statin therapy on type 2 diabetes risk evaluated at $a_D=1$ may be of particular clinical interest, quantifying the effect of assignment to the original statin therapy containing both components versus a modified treatment that removes the component possibly leading to weight gain.

### No isolation

**Fig. 5:**
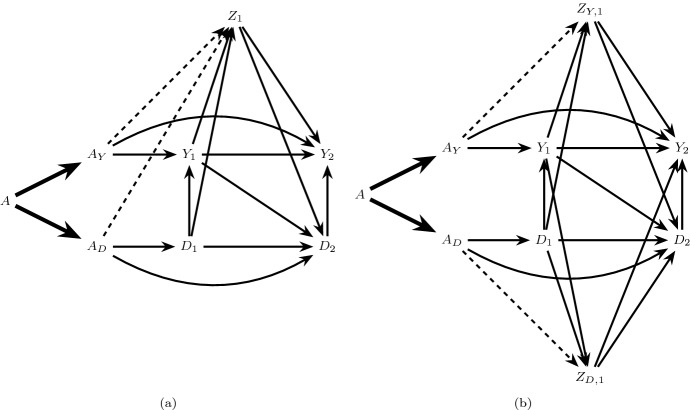
Causal graphs illustrating no isolation. **a** Violates $Z_k$ partition while **b** satisfies $Z_k$ partition

If there are direct arrows from $A_Y$ and $A_D$ into common causes of $Y_{k+1}$ and $D_{k+1}$, $k \in \{0,\ldots ,K\}$, as illustrated in Fig. 5, then both (6) and (7) fail. In this case, both the $A_Y$ separable effects (4) and the $A_D$ separable effects (5) quantify direct and indirect effects of the treatment on the event of interest, outside of and through, the competing event. When both conditions (6) and (7) fail, we will say there is *no isolation*.

There are two important cases of no isolation that have different implications for the interpretation of separable effects and, as we will see, their identification in a two-arm trial. First, suppose there are direct arrows from $A_Y$ and $A_D$ into the same set of common causes $Z_k$ of $\underline{Y}_{k+1}$ and $\underline{D}_{k+1}$, as illustrated in Fig. 5a. In this case, the $A_Y$ separable effects and the $A_D$ separable effects will capture common downstream pathways (e.g. $Z_1 \rightarrow Y_2$ in Fig. 5a) between the original treatment *A* and the event of interest $Y_{k+1}$.

Alternatively, suppose $A_Y$ and $A_D$ may only exert effects on *different* sets of common causes $Z_{Y,1}$ and $Z_{D,1}$ of $Y_{k+1}$ and $D_{k+1}$ as illustrated in Fig. 5b; here $A_Y$ exerts effects on $Y_{k+1}$ through one set of causal paths from $A_Y$ to $Y_{k+1}$, and $A_D$ exerts effects on $Y_{k+1}$ through a distinct set of causal paths. In this case, the $A_Y$ separable effects and the $A_D$ separable effects will capture no common pathways between the original treatment *A* and the event of interest $Y_{k+1}$.

### $Z_k$ partition

Suppose there exist vectors $Z_{D,k}, Z_{Y,k}$ such that $Z_k \equiv (Z_{D,k}, Z_{Y,k})$, $k>0$, and

8 $$\begin{aligned}&\text {The only causal paths from } A_Y \text { to } D_{k+1} \text { and } Z_{D,k+1}, k=0,...,K \text { are through } \nonumber \\&Y_{j} \text { or any component of } Z_{Y,j}, j=0,...,k. \end{aligned}$$

9 $$\begin{aligned}&\text {The only causal paths from } A_D \text { to } Y_{k+1} \text { and } Z_{Y,k+1}, k=0,...,K \text { are through } \nonumber \\&D_{j+1} \text { or any component of } Z_{D,j}, j=0,...,k. \end{aligned}$$

When both conditions (8) and (9) hold we will say there is a $Z_k$ *partition*.

**Fig. 6:**
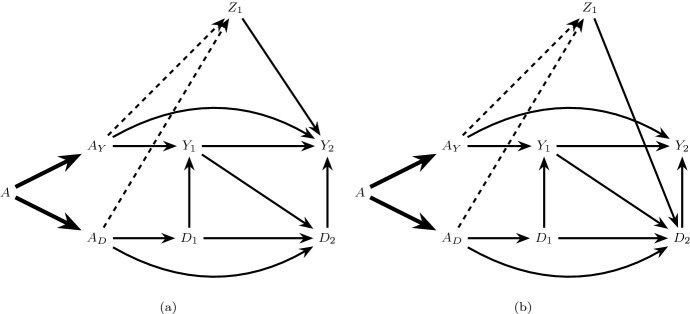
Causal graphs illustrating partial isolation but violation of $Z_k$ partition. $A_Y$ partial isolation holds in **a** and $A_D$ partial isolation holds in **b**

The assumption of a $Z_k$ partition holds trivially under full isolation for any partition of $Z_k$ as illustrated in Fig. 2b. However, this assumption will only hold in some cases of partial isolation (e.g. Fig. 4) and no isolation (e.g. Fig. 5b). $Z_k$ partition fails under the case of no isolation represented in Fig. 5a, which is the generalization of Robins and Richardson’s (2010) extended graph in Figure 6A to the time dependent case.

$Z_k$ partition also fails under the case of $A_Y$ partial isolation represented in Fig. 6a and $A_D$ partial isolation represented in Fig. 6b. Under any version of $Z_k$ partition, the $A_Y$ separable effects and the $A_D$ separable effects will capture no common pathways between the original treatment *A* and the event of interest $Y_{k+1}$.

## Identification of separable effects

**Fig. 7:**
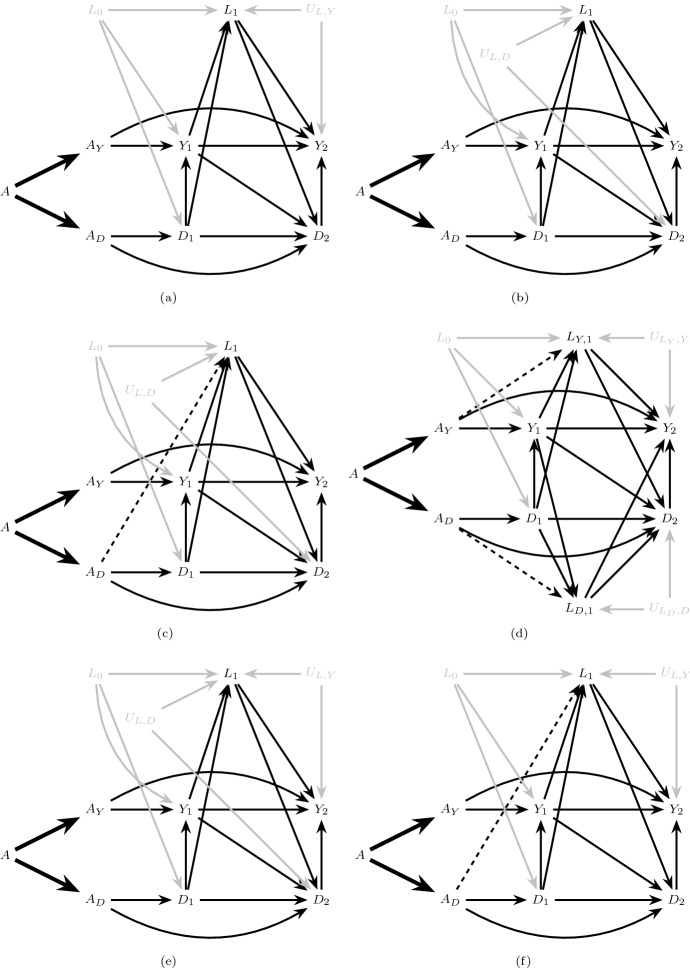
Extended graphs that explicitly depict measured and unmeasured variables. The dismissible component conditions hold in **a**–**d**. The dismissible component conditions are violated in **e**–**f**

Regardless of the isolation assumptions that impact the interpretation of separable effects, if we had data from a four-arm trial in which $A_Y$ and $A_D$ were randomly assigned with no loss to follow-up, we would be guaranteed identification of the separable effects (Stensrud et al. 2020; Robins 2016); that is, we could identify, for $k \in \{0,\ldots ,K\}$,

10 $$\begin{aligned}&\Pr (Y_{k+1}^{a_Y,a_D}=1) \text { for } a_Y,a_D \in \{0,1\} \end{aligned}$$

by $\Pr (Y_{k+1}=1 \mid A_Y = a_Y, A_D = a_D)$ (Hernan and Robins 2018). However, in order to identify (10) for $a_Y\ne a_D$ in the absence of a four-arm trial, we must make assumptions that are not guaranteed to hold, even in a two-armed trial such as that described in Sect. 2 with no loss to follow-up. In addition to the generalized decomposition assumption, consider the following assumptions that are expected to hold by design when *A* is randomly assigned (Hernan and Robins 2018) (recalling that $\overline{L}_k$ is the measured covariate history in our two-arm trial which may or may not coincide with $\overline{Z}_k$):

1. Exchangeability: 11 Figure 7 illustrates various extended graphs that explicitly depict measured (e.g. $L_0, L_1$) and unmeasured (e.g. $U_{L,Y}$) variables. Exchangeability is represented in Fig. 7a–f by the absence of any unblocked backdoor paths between *A* and $(\underline{Y}_{1},\underline{D}_{1},\underline{L}_{1}$) conditional on $L_0$ (Pearl 2009).
2. Consistency:
  12 $$\begin{aligned}&\!\!\!\!\! \text {If } A=a, \text { then } \bar{Y}_{k+1} = \bar{Y}^{a}_{k+1}, \bar{D}_{k+1} = \bar{D}^{a}_{k+1} \text { and } \bar{L}_{k+1} = \bar{L}^{a}_{k+1} \text { for } k \in \{0,\ldots ,K\}. \end{aligned}$$
  Consistency states that if an individual has observed treatment consistent with an intervention that sets $A=a$, then that individual’s future observed outcomes and time-varying covariates are equal to his/her counterfactual outcomes and time-varying covariates, respectively, under an intervention that sets $A=a$.
3. Positivity:
  13 $$\begin{aligned}&f_{L_0}(l_0)>0\implies \quad \Pr (A=a\mid L_0=l_0)>0, \text { for } a\in \{0,1\} \end{aligned}$$
  Assumption (13) states that, for any possibly observed level of the measured baseline covariates, there exist individuals with $A=1$ and individuals with $A=0$.

The above assumptions guarantee identification of the total effect (1), a contrast of $\Pr (Y_{k+1}^{a}=1)$ for different levels of *a* (Young et al. 2020) but are not sufficient for identification of separable effects, contrasts of $\Pr (Y_{k+1}^{a_Y,a_D}=1)$ for different levels of $a_Y$ and $a_D$ which require the following additional assumptions.

**Fig. 8:**
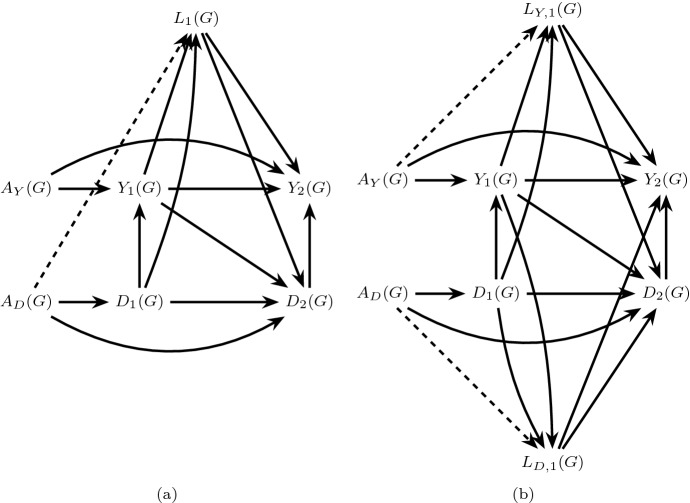
The graph in **a** is a successive transformation of Fig. 4a for $L_1=Z_1$ that represents a hypothetical trial *G* in which both $A_Y$ and $A_D$ are randomly assigned (We have removed $L_0$ to avoid clutter, but all our arguments are valid in the presence of $L_0$). The graph in **b** is a transformation of Fig. 5b, in which $L_{Y,1}(G) \equiv Z_{Y,1}(G), L_{D,1}(G) \equiv Z_{D,1}(G)$. All dismissible component conditions hold in both graphs

4. Dismissible component conditions:
  Let *G* refer to a hypothetical four-arm trial in which both $$A_Y$$ and $$A_D$$ are randomly assigned, possibly to different values; We add the string “(*G*)” to indicate the random variables that are defined in this trial. In particular, let $$Y_{k+1}(G)$$ and $$ D_{k+1}(G)$$ be the outcome of interest and the competing event had we, contrary to fact, randomly assigned $$A_Y(G)$$ and $$A_D(G)$$. Furthermore, let $$L_{Y,k}(G)$$ and $$L_{D,k}(G)$$ be disjoint vectors such that $$L_{k}(G) \equiv (L_{Y,k}(G),L_{D,k}(G))$$. We define the following conditions for $$k \in \{0,\ldots ,K\}$$: 14151617
  It follows directly from the generalized decomposition assumption that, using d-separation rules (Robins and Richardson 2010; Pearl 2009), the dismissible component conditions can be read off of a transformation of the extended causal DAG, representing an augmented version of our original data generating assumption, in which *A* and the deterministic arrows originating from *A* are eliminated. These transformations are isomorphic to dynamic Single World Intervention Graphs (SWIGs) (Richardson and Robins 2013; Robins et al. 2020), with interventions on $A_Y$ and $A_D$ (we have explicitly drawn such a SWIG in Appendix Fig. 11). See also similar results in Didelez (2018, Figure 2). We denote these graphical transformations as *G* transformations, describing a four-arm trial where $A_Y$ and $A_D$ are randomly assigned.
  For example, consider Fig. 8a, a transformation of Fig. 4a, simply assuming $L_k \equiv Z_k$. Assumption (14) holds in Fig. 8a by the absence of any unblocked backdoor paths between $A_D(G)$ and $Y_2(G)$ conditional on $A_Y(G)$, $D_1(G)$, $D_2(G)$, $L_1(G)$ and $Y_1(G)$, and similarly assumption (15) holds due to the absence of any unblocked paths between $A_Y(G)$ and $D_2(G)$ conditional on $A_D(G)$, $D_1(G)$, $L_1(G)$ and $Y_1(G)$. Analogously, by choosing $L_{k}(G) = (L_{Y,k}(G),\emptyset ),k=1,2$, (16) and (17) also hold in Fig. 8a.
  Consider also the examples in Fig. 7; under *G* transformations of each graph, all dismissible component conditions hold in Fig. 7a–d, where $L_{D,1}= L_1$ and $L_{Y,1}= \emptyset$ in Fig. 7a–c. By contrast, Fig. 7e–f illustrate failure of these conditions under their *G* transformations. For example, while (15)–(17) hold in Fig. 7e, (14) is violated by the the unblocked collider path $A_D(G) \rightarrow \boxed {D_2(G)} \leftarrow U_{L,D} \rightarrow \boxed {L_1(G)} \leftarrow U_{L,Y} \rightarrow Y_2(G)$, regardless of whether we define $L_{D,1}= L_1$ and $L_{Y,1}= \emptyset$ or $L_{Y,1}= L_1$ and $L_{D,1}= \emptyset$. Indeed, here $U_{L,D}$ and $U_{L,Y}$ are recanting districts (Shpitser 2013; Robins et al. 2020), and our identification conditions would hold if we were able to measure either $U_{L,D}$ or $U_{L,Y}$. Similarly, in Fig. 7f, while (15)–(17) hold when we define $L_{D,1}= L_1$ and $L_{Y,1}= \emptyset$, (14) is violated by the unblocked collider path $A_D(G) \rightarrow \boxed {L_1(G)} \leftarrow U_{L,Y} \rightarrow Y_2(G)$.
5. Strong positivity:
  18 $$\begin{aligned}&f_{\overline{L}_k,D_{k+1},Y_k}(\overline{l}_k,0,0)> 0 \implies \quad \Pr (A=a|D_{k+1}=Y_k=0,\overline{L}_k=\overline{l}_k)>0, \nonumber \\&\text {for } a\in \{0,1\}\text { and }k \in \{0,\ldots ,K\}. \end{aligned}$$
  Assumption (18) implies (13) and requires that for any possibly observed level of the measured time-varying covariate history among those surviving all events through each follow-up time, there exist individuals with $A=1$ and individuals with $A=0$. Even when *A* is randomized, assumption (18) does not hold by design. However, it can be assessed in the observed data. Given the dismissible component conditions, we need assumption (18) to ensure that all the terms in the identification formula are well-defined (see Sect. 6.2).

The identification conditions in this section are linked to previous general identification results on identification of path-specific effects (Shpitser 2013; Avin et al. 2005) (who did not consider competing events): there exist (cross-world) path specific effects that may be identified by isomorphic identification formulas as the separable effects (Shpitser 2013).

### Relation between isolation and dismissible component conditions

Note that $Z_k$ partition is a *necessary* condition for the dismissible component conditions to hold for any choice of measured covariates $L_k$ and their partition (see proof in Appendix C). However, $Z_k$ partition is not *sufficient* to ensure these conditions as also illustrated by Fig. 7. For example, in Fig. 7e, full isolation holds but, as we noted above, the dismissible component conditions fail due to failure to measure either the common cause $U_{L,D}$ or $U_{L,Y}$. Similarly, the graph in Fig. 7f satisfies $Z_k$ partition, but, as we noted above, the dismissible component conditions fail due to failure to measure the common cause $U_{L,Y}$.

In Appendix C we also show that: (i) if the dismissible component conditions hold when we define $L_{D,k}=L_k$ and $L_{Y,k}=\emptyset$ for all $k\in \{1,\dots ,K\}$, then $A_Y$ partial isolation holds; (ii) if the dismissible component conditions hold when we define $L_{Y,k}=L_k$ and $L_{D,k}=\emptyset$ for all $k\in \{1,\dots ,K\}$, then $A_D$ partial isolation holds; and (iii) if the dismissible component conditions hold when we choose either of the partitions in (i) and (ii) then full isolation holds and $L_k$ is independent of *A* at any *k*, given the measured past.

More generally, our identification conditions will only hold when $L_{D,k}$ and $L_{Y,k}$ are independent of each other given the measured past. When both $L_{D,k}$ and $L_{Y,k}$ are non-empty, our identification results are related to Robins and Richardson’s (2010) identification results for mediation estimands in a non-extended DAG with a recanting witness under cross-world independence assumptions (See Robins and Richardson’s 2010 Figure 2b and Section 4.4).

### The g-formula for separable effects

For $k \in \{0,\ldots ,K\}$, let $l_k = (l_{Y,k},l_{D,k})$ be a realization of the measured time-varying covariates at *k*, such that $l_{Y,k}$ and $l_{D,k}$ are possible realizations of $L_{Y,k}$ and $L_{D,k}$, respectively (a chosen partition of $L_k$ under an assumed temporal order $L_{D,k}, L_{Y,k}$). Provided that exchangeability, consistency, positivity and the 4 dismissible component conditions hold, we can identify $\Pr (Y^{a_Y, a_D}_{k+1}=1)$ by

19 $$\begin{aligned}&\sum _{\bar{l}_k} \Big [ \sum _{s=0}^{k} \Pr (Y_{s+1}=1 \mid D_{s+1}= Y_{s}=0, \bar{L}_{s} = \bar{l}_{s}, A = a_Y) \nonumber \\&\prod _{j=0}^{s} \big \{ \Pr (D_{j+1}=0 \mid D_{j}= Y_{j}=0, \bar{L}_{j} = \bar{l}_{j}, A = a_D) \nonumber \\&\quad \times \Pr (Y_{j}=0 \mid Y_{j-1}=D_{j}=0, \bar{L}_{j-1} = \bar{l}_{j-1}, A = a_Y) \nonumber \\&\quad \times f(L_{Y,j}=l_{Y,j} \mid Y_{j} = D_{j} = 0, \bar{L}_{j-1}=\bar{l}_{j-1}, L_{D,j}= l_{D,j}, A = a_Y) \nonumber \\&\quad \times f(L_{D,j}=l_{D,j} \mid Y_{j} = D_{j} = 0, \bar{L}_{j-1}=\bar{l}_{j-1}, A = a_D) \big \} \Big ], \end{aligned}$$

$k \in \{0,\ldots ,K\}$, where for any vector of random variables *A* and *B*, $f(A = a \mid B= b)$ is the conditional density of *A* given *B* evaluated at *a*, *b*. See Appendix B for proof. We will refer to expression (19) as the *g-formula* (Robins 1986) for $\Pr (Y^{a_Y, a_D}_{k+1}=1)$, which is equivalent to identification formulas for path-specific effects with a different interpretation and under different identification assumptions (Shpitser 2013) and interventionist’s mediation effects (Robins et al. 2020).

### The g-formula in the presence of censoring

We now relax the assumption of no losses to follow-up, allowing that some individuals are censored at some point during the study. For $k>0$, let $C_{k}$ denote censoring by loss to follow-up by interval *k*, and assume a temporal order $(C_{k},D_{k},Y_{k},L_{k})$ in each interval $k > 0$. We remind the reader that the the temporal ordering assumption is analogous to assumptions about ties in continuous time settings, which becomes practically irrelevant when the time intervals are small. Hereby, we will implicitly redefine all counterfactual outcomes $Y_{k+1}^{a_Y, a_D}$ in terms of outcomes under an additional intervention that eliminates censoring.

When censoring is present, the isolation conditions defined in Sect. 5 and their implications for interpretation of separable effects are unchanged. However, in this case, additional exchangeability, positivity and consistency assumptions are required for identification of (10) using only the observed data. Given assumptions (28)–(37) in Appendix B, which extend the assumptions of Sect. 6 to allow that censoring is present and dependent on the measured time-varying risk factors $\overline{L}_k$, we can identify (10) by

20 $$\begin{aligned}&\sum _{\bar{l}_k} \Big [ \sum _{s=0}^{k} \Pr (Y_{s+1}=1 \mid C_{s+1}= D_{s+1}= Y_{s}=0,\bar{L}_{s} = \bar{l}_{s}, A=a_Y) \nonumber \\&\prod _{j=0}^{s} \big \{ \Pr (D_{j+1}=0 \mid C_{j+1}=D_{j}= Y_{j}=0, \bar{L}_{j} = \bar{l}_{j}, A = a_D) \nonumber \\&\quad \times \Pr (Y_{j}=0 \mid C_{j}=D_{j}= Y_{j-1}=0,\bar{L}_{j-1} = \bar{l}_{j-1}, A = a_Y) \nonumber \\&\quad \times f(L_{Y,j}=l_{Y,j} \mid C_{j}= Y_{j} = D_{j} = 0, \bar{L}_{j-1} = \bar{l}_{j-1}, L_{D,j}= l_{D,j}, A = a_Y) \nonumber \\&\quad \times f(L_{D,j}=l_{D,j} \mid C_{j}= Y_{j} = D_{j} = 0, L_{j-1}=\bar{l}_{j-1}, A = a_D) \big \} \Big ]. \end{aligned}$$

See Appendix B for proof. We say expression (20) is the g-formula for (10) under elimination of censoring. When assumptions (28)–(37) hold replacing $\overline{L}_k = L_0$, $k \in \{0,\ldots ,K\}$ then identification of (10) is achieved by a simplified version of (20), which was given in Stensrud et al. (2020).

## Estimation of separable effects and data example

The g-formula (20) has the following alternative representations,

21 $$\begin{aligned} \sum _{s=0}^{k} E&[ W_{C,s} W_{D,s} W_{L_{D},s} (1-Y_{s}) (1-D_{s+1}) Y_{s+1} \mid A=a_Y], \end{aligned}$$

where

$$\begin{aligned} W_{D,s}&= \frac{\prod _{j=0}^{s} \Pr (D_{j+1}=0 \mid C_{j+1}=D_{j}= Y_{j}=0, \bar{L}_{j}, A = a_D) }{ \prod _{j=0}^{s} \Pr (D_{j+1}=0 \mid C_{j+1}=D_{j}= Y_{j}=0, \bar{L}_{j}, A = a_Y) }, \\ W_{L_{D},s}&= \frac{\prod _{j=0}^{s} \Pr (A =a_D \mid C_{j}= Y_{j} = D_{j} = 0, L_{D,j}, \bar{L}_{j-1}) }{ \prod _{j=0}^{s} \Pr (A =a_Y \mid C_{j}= Y_{j} = D_{j} = 0, L_{D,j}, \bar{L}_{j-1}) } \\&\quad \times \frac{\prod _{j=0}^{s} \Pr (A =a_Y \mid C_{j}= Y_{j} = D_{j} = 0, \bar{L}_{j-1}) }{ \prod _{j=0}^{s} \Pr (A =a_D \mid C_{j}= Y_{j} = D_{j} = 0, \bar{L}_{j-1}) }, \\ W_{C,s}&= \frac{I(C_{s+1} =0) }{ \prod _{j=0}^{s} \Pr (C_{j+1}=0 \mid C_{j}=D_{j}= Y_{j}=0, \bar{L}_{j}, A) }, \end{aligned}$$

and

22 $$\begin{aligned} \sum _{s=0}^{k} E&\{ W_{C,s} W_{Y,s} W_{L_{Y},s} (1-Y_{s}) (1-D_{s+1}) Y_{s+1} \mid A=a_D \}, \end{aligned}$$

where $W_{C,s}$ is defined as in (21) and

$$\begin{aligned} W_{Y,s}&= \frac{ \Pr (Y_{s+1}=1 \mid C_{s+1}=D_{s+1}= Y_{s}=0, \bar{L}_{s}, A = a_Y) }{\Pr (Y_{s+1}=1 \mid C_{s+1}=D_{s+1}= Y_{s}=0, \bar{L}_{s}, A = a_D) } \\&\quad \times \frac{\prod _{j=0}^{s-1} \Pr (Y_{j+1}=0 \mid C_{j+1}=D_{j+1}= Y_{j}=0, \bar{L}_{j}, A = a_Y) }{ \prod _{j=0}^{s-1} \Pr (Y_{j+1}=0 \mid C_{j+1}=D_{j+1}= Y_{j}=0, \bar{L}_{j}, A = a_D) }, \\ W_{L_{Y},s}&= \frac{\prod _{j=0}^{s} \Pr (A =a_Y \mid C_{j}= Y_{j} = D_{j} = 0, \bar{L}_{j}) }{ \prod _{j=0}^{s} \Pr (A =a_D \mid C_{j}= Y_{j} = D_{j} = 0, \bar{L}_{j}) } \\&\quad \times \frac{\prod _{j=0}^{s} \Pr (A =a_D \mid C_{j}= Y_{j} = D_{j} = 0, L_{D,j}, \bar{L}_{j-1}) }{ \prod _{j=0}^{s} \Pr (A =a_Y \mid C_{j}= Y_{j} = D_{j} = 0, L_{D,j}, \bar{L}_{j-1}) }, \end{aligned}$$

as formally shown in Appendix D.

Here, $$W_{Y,s} $$ and $$W_{D,s} $$ are products of conditional discrete cause-specific hazards of the event of interest and the competing event, respectively. The weights $$W_{L_{Y},s} $$ and $$W_{L_{D},s} $$ are derived from re-expression of conditional probabilities of $$L_{Y,s}$$ and $$L_{D,s}$$, respectively, see Appendix D.

Representations (21) and (22) motivate weighted estimators of the separable effects, which generalize the weighted estimators given by Stensrud et al. (2020) and are related to inverse odds ratio weights for mediation analysis (Tchetgen Tchetgen 2013). We let $\nu _{k}$ denote (20), and $(\overline{L}_{k,i},L_{D,k,i})$ is individual *i*’s values of $(\overline{L}_{k},L_{D,k})$ for a user-chosen partition of $L_k$.

Define

$$\begin{aligned} \hat{W}_{D,k,i} (\hat{\beta }_D)&= \frac{\prod _{j=0}^{k} \Pr (D_{j+1}=0 \mid C_{j+1}=D_{j}= Y_{j}=0, \bar{L}_{j,i}, A = a_D; \hat{\beta }_D) }{ \prod _{j=0}^{k} \Pr (D_{j+1}=0 \mid C_{j+1}=D_{j}= Y_{j}=0, \bar{L}_{j,i}, A = a_Y ; \hat{\beta }_D) }, \\ \hat{W}_{L_{D},k,i} (\hat{\beta }_{L1},\hat{\beta }_{L2})&= \frac{\prod _{j=0}^{k} \Pr (A =a_D \mid C_{j}= Y_{j} = D_{j} = 0, L_{D,j,i}, \bar{L}_{j-1,i};\hat{\beta }_{L1}) }{ \prod _{j=0}^{k} \Pr (A =a_Y \mid C_{j}= Y_{j} = D_{j} = 0, L_{D,j,i}, \bar{L}_{j-1,i}; \hat{\beta }_{L1}) } \\&\quad \times \frac{\prod _{j=0}^{k} \Pr (A =a_Y \mid C_{j}= Y_{j} = D_{j} = 0, \bar{L}_{j-1,i}; \hat{\beta }_{L2}) }{ \prod _{j=0}^{k} \Pr (A =a_D \mid C_{j}= Y_{j} = D_{j} = 0, \bar{L}_{j-1,i}; \hat{\beta }_{L2}) }, \\ \hat{W}_{C,k,i} (\hat{\beta }_{C})&= \frac{I(C_{k+1} =0) }{ \prod _{j=0}^{k} \Pr (C_{j+1}=0 \mid C_{j}=D_{j}= Y_{j}=0, \bar{L}_{j,i}, A_{i}; \hat{\beta }_{C}) }, \end{aligned}$$

where $\Pr (D_{j+1}=0 \mid C_{j+1}=D_{j}= Y_{j}=0, \bar{L}_{j}, A = a_D; \beta _D)$ is a parametric model for the numerator (and denominator) of $W_{D,k}$ indexed by parameter $\beta _D$, and $\hat{\beta }_D$ is a consistent estimator of $\beta _D$ (e.g. the MLE). The terms in $\hat{W}_{L_{D},k,i} (\hat{\beta }_{L1},\hat{\beta }_{L2})$ and $\hat{W}_{C,k,i} (\hat{\beta }_{C})$ are defined analogously, where $\hat{\beta }_{L1},\hat{\beta }_{L2},\hat{\beta }_{C}$ are consistent estimators of corresponding model parameters $\beta _{L1 },\beta _{L2 },\beta _{C}$, respectively.

Let $\beta _1 = (\beta _D, \beta _{L1 },\beta _{L2 },\beta _{C})$, and define the estimator $\hat{\nu }_{1,k}$ of $\nu _{k}$ as the solution to the estimating equation $\sum _{i=1}^{n}U_{1,k,i}(\nu _{k},\hat{\beta }_1)=0$ with respect to $\nu _{k}$ with

$$\begin{aligned}&U_{1,k,i}(\nu _{k},\hat{\beta }_1) =&I(A_i=a_Y) \Big [ \sum _{s=0}^{k} \{ \hat{W}_{1,s,i}(\hat{\beta }_1) Y_{s+1,i} (1-Y_{s,i}) (1-D_{s+1,i}) \} - \nu _{k} \Big ], \end{aligned}$$

and $$\hat{W}_{1,s,i}( \hat{\beta }_1) = \hat{W}_{D,s,i} (\hat{\beta }_{D}) \hat{W}_{L_{D},s,i} (\hat{\beta }_{L1},\hat{\beta }_{L2}) \hat{W}_{C,s,i} (\hat{\beta }_{C}) $$.

Provided that the models indexed by elements in $\beta _1$ are correctly specified and $\hat{\beta }_1$ is a consistent estimator for $\beta _1$, then consistency of $\hat{\nu }_{1, k}$ for $\nu _{k}$ follows because (20) and (21) are equal. We describe an implementation algorithm for $\hat{\nu }_{1, k}$ in Appendix E. In practice, we can use popular regression models for binary outcomes to estimate the weights $W_{D,k}$ and $W_{C,k}$. However, when we parameterize the terms in $\hat{W}_{L_{D},k} (\hat{\beta }_{L1},\hat{\beta }_{L2})$, we must ensure that the statistical models are congenial, which may fail for popular models, such as logistic regressions models. In Appendix D, we have provided an alternative expression of $W_{L_{Y},k}$ that motivates different weighted estimators based on estimation of the conditional joint densities of $L_k$. These weighted estimators avoid the problem of incongenial models at the expense of needing to model higher dimensional quantities.

The estimator based on (22) is derived analogously to the estimator based on (21). Suppose

$$\begin{aligned} \hat{W}_{Y,k,i} (\hat{\beta }_{Y})&= \frac{ \Pr (Y_{k+1}=1 \mid C_{k+1}=D_{k+1}= Y_{k}=0, \bar{L}_{k,i}, A = a_Y; \hat{\beta }_{Y}) }{\Pr (Y_{k+1}=1 \mid C_{j+1}=D_{k+1}= Y_{k}=0, \bar{L}_{k,i}, A = a_D; \hat{\beta }_{Y}) } \\&\quad \times \frac{\prod _{j=0}^{k-1} \Pr (Y_{j+1}=0 \mid C_{j+1}=D_{j+1}= Y_{j}=0, \bar{L}_{j,i}, A = a_Y; \hat{\beta }_{L3}) }{ \prod _{j=0}^{k-1} \Pr (Y_{j+1}=0 \mid C_{j+1}=D_{j+1}= Y_{j}=0, \bar{L}_{j,i}, A = a_D; \hat{\beta }_{L3}) }, \\ \hat{W}_{L_{Y},k,i} (\hat{\beta }_{L3},\hat{\beta }_{L4})&= \frac{\prod _{j=0}^{k} \Pr (A =a_Y \mid C_{j}= Y_{j} = D_{j} = 0, \bar{L}_{j,i}; \hat{\beta }_{L4}) }{ \prod _{j=0}^{k} \Pr (A =a_D \mid C_{j}= Y_{j} = D_{j} = 0, \bar{L}_{j,i}; \hat{\beta }_{L4}) } \\&\quad \times \frac{\prod _{j=0}^{k} \Pr (A =a_D \mid C_{j}= Y_{j} = D_{j} = 0, L_{D,j,i}, \bar{L}_{j-1,i}; \hat{\beta }_{C}) }{ \prod _{j=0}^{k} \Pr (A =a_Y \mid C_{j}= Y_{j} = D_{j} = 0, L_{D,j,i}, \bar{L}_{j,i}; \hat{\beta }_{C}) }, \end{aligned}$$

where the terms in $\hat{W}_{Y,k,i} (\hat{\beta }_{Y})$, $\hat{W}_{L_{Y},k,i} (\hat{\beta }_{L3},\hat{\beta }_{L4})$ are statistical models for binary outcomes, and where $\hat{\beta }_{Y}, \hat{\beta }_{L3},\hat{\beta }_{L4}$ are consistent estimators for $\beta _Y,\beta _{L3 },\beta _{L4 }$, respectively. Similar to $\hat{W}_{L_{D},k,i} (\hat{\beta }_{L1},\hat{\beta }_{L2})$, however, we must ensure that congenial models are used to estimate the terms in $\hat{W}_{L_{Y},k,i} (\hat{\beta }_{L3},\hat{\beta }_{L4})$.

Let $\beta _2 = (\beta _Y, \beta _{L3},\beta _{L4},\beta _{C})$, and define the estimator $\hat{\nu }_{2,k}$ of $\nu _{k}$ as the solution to the estimating equation $\sum _{i=1}^{n}U_{2,k,i}(\nu _{k},\hat{\beta }_2)=0$ with respect to $\nu _{k}$, where

$$\begin{aligned}&U_{2,k,i}(\nu _{k},\hat{\beta }_2) = I(A_i=a_D) \Big [ \sum _{s=0}^{k} \{ \hat{W}_{2,s,i}(\hat{\beta }_{2}) Y_{s+1,i} (1-Y_{s,i}) (1-D_{s+1,i}) \} - \nu _{k} \Big ], \end{aligned}$$

and $\hat{W}_{2,s,i}(\hat{\beta }_{2} ) = \hat{W}_{C,s,i} (\hat{\beta }_{C}) \hat{W}_{Y,s,i} (\hat{\beta }_{Y})\hat{W}_{L_{Y},s,i} (\hat{\beta }_{L3},\hat{\beta }_{L4})$. Analogous to the estimator based on (21), provided that the models indexed by elements in $\beta _2$ are correctly specified and $\hat{\beta }_2$ is a consistent estimator for $\beta _2$, then consistency of $\hat{\nu }_{2,k}$ for $\nu _{k}$ follows because (20) and (22) are equal.

### Simplified estimators under assumptions on $L_k$

Given a user-chosen partition of $L_k$ such that $L_{Y,k} \equiv L_k, L_{D,k} \equiv \emptyset$ for $k = 0,\dots , K$, then $W_{L_{D},k} =1$ and the consistency of $\hat{\nu }_{1,k}$ only requires consistent estimation of the weights $W_{D,k}$ and $W_{C,k}$. Thus, if the identification conditions hold and there is no direct effect (arrow) from $A_D$ to $L_k$, which implies that $A_D$ partial isolation holds (see Lemma 6), we suggest using the estimator $\hat{\nu }_{1,k}$, which is motivated by (21), because it does not require any modelling of the covariate distributions ($\overline{L}_k$).

Similarly, the partition $L_{D,k} \equiv L_k, L_{Y,k} \equiv \emptyset$ gives $W_{L_{Y},k} =1$, such that the consistency of $\hat{\nu }_{2,k}$ only relies on consistent estimation of the weights $W_{Y,k}$ and $W_{C,k}$. Thus, if the identification conditions hold and there is no direct effect (arrow) from $A_Y$ to $L_k$, which implies $A_Y$ partial isolation holds (see Lemma 5), we suggest using the estimator $\hat{\nu }_{2,k}$, which is motivated by (22).

Of course, these simplified $L_k$ partitions are only justified if they satisfy the dismissible component conditions. As discussed in Sect. 6.1, identification under these simplified $L_k$ partitions implies partial or full isolation, impacting the interpretation of the separable effects.

### Data example: blood pressure therapy and acute kidney injury

As an illustration, we analyzed data from the Systolic Blood Pressure Intervention Trial (SPRINT) (SPRINT Research Group 2015), which randomly assigned individuals to intensive ($A=1$) or standard ($A=0$) blood pressure treatment. We used follow-up data from each month $k+1$, $k=0\ldots ,29$ and restricted our analysis to participants aged older than 75 years at baseline in whom the most deaths (competing events) occurred (Williamson et al. 2016). For simplicity, we further restricted to those patients with complete data on baseline covariates (described below). This resulted in a data set with 1304 and 1297 in the intensive ($A=1$) and standard ($A=0$) blood pressure therapy arms, respectively. During the 30-month follow-up period, 107 and 98 of these patients were lost to follow-up (censored) in some month $k+1\le 30$ in the intensive and standard arms, respectively.

In order to adjust for informative censoring by loss to follow-up, we used inverse probability of censoring weighted Aalen-Johansen estimators (Aalen and Johansen 1978; Young et al. 2020) to estimate the total effects of treatment assignment on the cause-specific cumulative incidences at each $k+1$ of kidney injury and mortality. We adjusted for the baseline covariates ($L_0$) smoking status, history of clinical or subclinical cardiovascular disease, clinical of subclinical chronic kidney disease, statin use and gender as well as the time-varying covariates ($L_k$) defined by the most recent measurements of systolic and diastolic blood pressure, scheduled monthly for the first 3 months and every 3 months thereafter. The weight denominators were estimated under the following pooled logistic model for the probability of being censored within each month $k+1$ given the measured past,

23 $$\begin{aligned}&\text {logit} \{\Pr (C_{k+1}=1 \mid D_{k}= Y_{k}=\bar{C}_{k}=0,A, \bar{L}_k)\} \end{aligned}$$

24 $$\begin{aligned}&\quad = \beta _{C,0,k+1} + \beta _{C,1}A + \beta _{C,2}Ak + \beta '_{C,1}L_0 + \beta '_{C,2}L_k, \end{aligned}$$

where $\beta _{C,0,k+1}$ are time-varying intercepts modeled as 3rd degree polynomials. For all analyses, 95% percent confidence intervals were constructed using 500 nonparametric bootstrap samples.

**Fig. 9:**
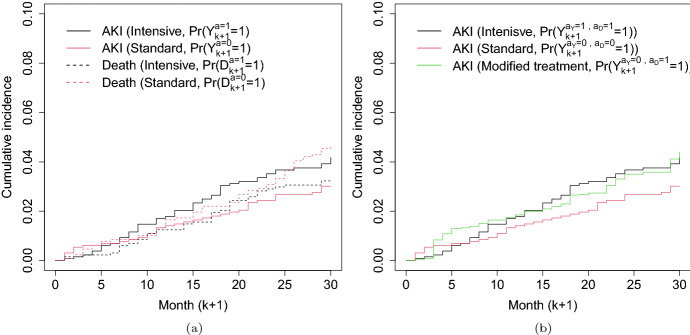
**a** Weighted Aalen-Johansen estimates of the cumulative incidence functions for acute kidney injury (AKI, solid lines) and death (dashed lines) under intensive ($a=1$, black) and standard ($a=0$, red) treatment. **b** Estimates of AKI cumulative incidence based on methods of Sect. 7 under a modified treatment containing only the $A_D$ component ($a_D=1,a_Y=0$, green). Cumulative incidence estimates under the original intensive ($a=a_Y=a_D=1$, black) and the standard ($a=a_Y=a_D=0$, red) of **a** are overlaid

The estimated cumulative incidence of acute kidney injury (the event of interest) under the intensive treatment assignment was consistently higher compared to standard treatment assignment (Fig. 9a, solid lines), in line with a harmful total effect on acute kidney injury. Specifically, the total effect estimate (on the additive scale) of intensive therapy assignment versus standard was 0.01 (95% CI: [0.00, 0.03]) at 30 months of follow-up. This weighted estimator is consistent for the total effect under weaker conditions than those outlined above for separable effects – unlike the separable effects, covariate adjustment here is only necessary due to censoring by loss to follow-up (Young et al. 2020). However, as discussed in Sect. 3, this harmful effect is hard to interpret due to a possible protective effect of intensive treatment assignment on death (the competing event). This concern is not easily ruled out by the data; the cumulative incidence of death under intensive treatment assignment is consistently slightly lower compared to standard treatment assignment over the 30-month follow-up with differences increasing at 25 months, as shown with dashed lines in Fig. 9a. At 30 months, the total effect estimate on mortality was -0.01 (95% CI: $[-0.03, 0.00]$). Effects that quantify mechanism are therefore naturally of interest.

As discussed in Sect. 5.2, for $A_Y$ defined as the component of treatment *A* that may exert biological effects on the kidneys, e.g. by relaxing the efferent arterioles, and $A_D$ defined as all remaining components of *A*, $A_Y$ partial isolation may be a reasonable assumption given background subject matter knowledge. Under this assumption, the $A_Y$ separable effect (4) evaluated at $a_D=1$ does not capture effects of the treatment on the competing event. It also may be of clinical interest as it quantifies the effect of removing the possibly harmful $A_Y$ component from the original treatment *A*.

Following the recommendations of Sect. 7.1, given our subject-matter driven assumption of $$A_Y$$ partial isolation, the inverse probability weighted estimator $$\hat{\nu }_{2,k}$$ described in Sect. 7, based on the representation (22), will rely on considerably fewer parametric model assumptions than the alternative estimator $$\hat{\nu }_{1,k}$$, based on the representation (21). The estimator $$\hat{\nu }_{2,k}$$ further avoids the problem of model incongeniality of $$\hat{\nu }_{1,k}$$ because we do not need to specify the weights $$W_{L_{Y},s} $$. Therefore, we used $$\hat{\nu }_{2,k}$$ to estimate (10) and, in turn, the $$A_Y$$ separable effect (4) evaluated at $$a_D=1$$ on acute kidney injury at each $$k+1$$ under the assumption that the measured baseline and time-varying covariates are sufficient to ensure identification, i.e. to adjust for common causes of the event of interest and the competing event. That is, we assume that the dismissible component conditions (14)–(17) hold under the partitioning $$L_k=L_{D,k}$$. This assumption, at best, approximately holds because $$L_k$$ contains only intermittent measurements of systolic and diastolic blood pressure.

We estimated $W_{Y,k}$ under the pooled logistic models

25 $$\begin{aligned}&\text {logit} \{\Pr (Y_{k+1}=1 \mid D_{k+1}= Y_{k+1} =\bar{C}_{k+1}=0,A=0, \bar{L}_k) \} \nonumber \\&\quad = \beta _{Y,0,k+1} + \beta '_{Y,1}L_0 + \beta '_{Y,2}L_k + \beta '_{Y,3}L_k^2 +\beta '_{Y,4}L_k k, \nonumber \\&\text {logit} \{ \Pr (Y_{k+1}=1 \mid D_{k+1}= Y_{k+1} =\bar{C}_{k+1}=0,A=1, \bar{L}_k) \} \nonumber \\&\quad = \beta _{Y,5,k+1} + \beta '_{Y,6}L_0 + \beta '_{Y,7}L_k + \beta '_{Y,8}L_k^2 + \beta '_{Y,9}L_k k, \nonumber \\ \end{aligned}$$

for $k = 0,\dots ,20$, where $\beta _{Y,0,k+1}$ and $\beta _{Y,5,k+1}$ are time-varying intercepts modeled as 3rd degree polynomials. The inverse probability of censoring weights $W_{C}$ were estimated under (24).

Figure 9b shows estimates of the counterfactual cumulative incidence for acute kidney injury under assignment to different combinations of $a_Y$ and $a_D$ over time. The vertical distance between the black and the green line is a point estimate of the additive $A_Y$ separable effect when $a_D=1$, and similarly the vertical distance between the red and the green line is the $A_D$ separable effect when $a_Y=0$. In particular, the estimated $A_Y$ separable effect of 0.00 (95% CI: $[-0.10, 0.02]$) at 30 months when $a_D=1$, suggests that removing the $A_Y$ component from the intensive therapy will not decrease the average risk of kidney injury at 30 months. R code is provided in the supplementary materials.

As a comparison, we estimated the cause-specific hazard ratio under a proportional hazards model conditional on the same baseline covariates ($L_0$) and time-varying covariates ($\overline{L}_k$), which was $1.23 (95\%: [0.82,1.93])$. This estimated association, which is conditional not only on acute kidney injury not having occurred before *k*, but also on death not having occurred before *k* and time-varying history of blood pressure measurements, is further from the null than our separable effect estimates. Importantly, this estimand is not guaranteed a causal interpretation even under our identifying conditions of Sect. 6 (Young et al. 2020; Stensrud et al. 2020).

### Sensitivity analysis

To illustrate a sensitivity analysis technique for violations of the dismissible component conditions, consider a selection bias function for dismissible component condition (14),

$$\begin{aligned} t_k(\bar{l}_k, a_Y) =&\Pr (Y^{a_Y,a_D=0,\bar{c}= 0}_{k+1}=1 \mid D^{a_Y,a_D=0,\bar{c}= 0}_{k+1}= Y^{a_Y,a_D=0,\bar{c}= 0}_{k}=0, \bar{L}^{a_Y,a_D=0,\bar{c}= 0}_{k} = \bar{l}_{k}) \\&- \Pr (Y^{a_Y,a_D=1,\bar{c}= 0}_{k+1}=1 \mid D^{a_Y,a_D=1,\bar{c}= 0}_{k+1}= Y^{a_Y,a_D=1,\bar{c}= 0}_{k}=0, \bar{L}^{a_Y,a_D=1,\bar{c}= 0}_{k} = \bar{l}_{k}), \end{aligned}$$

which is identified in a perfectly executed randomized trial in which $A_Y$ and $A_D$ are randomly assigned. Analogous sensitivity functions could be defined for dismissible component conditions (15)–(17). If dismissible component condition (14) holds for $\bar{L}_k$, we know that $t(\bar{L}_k, a_Y) = 0$. However, if (14) was violated, we would expect that $t_k(\bar{l}_k, a_Y) \ne 0$ for some values of $\bar{l}_k$ and $a_Y$. In particular, (14) can be violated in the presence of an unmeasured cause of $Y_k$ and $D_j$, where $0 < j \le k$.

While the following strategy for sensitivity analysis is applicable to any setting in which $Z_k$ partition holds, we consider a simpler setting in which (i) $A_Y$ partial isolation holds, (ii) dismissible component condition (14) holds for some $L' \equiv L'_D$ that contains the measured variable *L* as a subset, $L \subset L'$, and (iii) dismissible component conditions (15)–(17) hold. This is coherent with our blood pressure example in Sect. 7.2, and one such setting is described in Fig. 7f where (14) is violated due to failure of measuring $U_{L,Y}$. Now, suppose that $t_k(\bar{l}_k, a_Y)$ is known. Then the separable effects can be identified through the modified version of identification formula (22),

26 $$\begin{aligned} \sum _{s=0}^{k} E&\{ W_{C,s} W^{\dagger }_{Y,s} (1-Y_{s}) (1-D_{s+1}) Y_{s+1} \mid A=a_D \}, \end{aligned}$$

where

$$\begin{aligned} W^{\dagger }_{Y,s}&= \frac{ (-1)^{a_D} t_{s+1}(\bar{l}_{s+1}, a_Y) + \Pr (Y_{s+1}=1 \mid C_{s+1}=D_{s+1}= Y_{s}=0, \bar{L}_{s}, A = a_Y) }{\Pr (Y_{s+1}=1 \mid C_{s+1}=D_{s+1}= Y_{s}=0, \bar{L}_{s}, A = a_D) } \\&\quad \times \frac{\prod _{j=0}^{s-1} (-1)^{a_D} t_j(\bar{l}_j, a_Y) + \Pr (Y_{j+1}=0 \mid C_{j+1}=D_{j+1}= Y_{j}=0, \bar{L}_{j}, A = a_Y) }{ \prod _{j=0}^{s-1} \Pr (Y_{j+1}=0 \mid C_{j+1}=D_{j+1}= Y_{j}=0, \bar{L}_{j}, A = a_D) }, \\ \end{aligned}$$

which is equal to (22) under $A_Y$ partial isolation when $t_k(\bar{l}_k, a_Y)=0$ and $a_Y \ne a_D$ for all *k* and $\bar{l}_k$. Formula (26) motivates the estimator $\hat{\nu }^{\dagger }_{2,k}$, a modified version of $\hat{\nu }_{2,k}$ from Sect. 7, such that $\hat{\nu }^{\dagger }_{2,k}$ is the solution to the estimating equation $\sum _{i=1}^{n}U^{\dagger }_{2,k,i}(\nu _{k},\hat{\beta }_2)=0$ with respect to $\nu _{k}$, where

$$\begin{aligned}&U^{\dagger }_{2,k,i}(\nu _{k},\hat{\beta }_2) \\&\quad = I(A_i=a_D) \Big [ \sum _{s=0}^{k} \{ \hat{W}^{\dagger }_{2,s,i}(\hat{\beta }_{2}) Y_{s+1,i} (1-Y_{s,i}) (1-D_{s+1,i}) \} - \nu _{k} \Big ], \end{aligned}$$

and $$\hat{W}^{\dagger }_{2,s,i}(\hat{\beta }_{2} ) = \hat{W}_{C,s,i} (\hat{\beta }_{C}) \hat{W}^{\dagger }_{Y,s,i} (\hat{\beta }_{Y})$$, where

$$\begin{aligned}&\hat{W}^{\dagger }_{Y,k,i} (\hat{\beta }_{Y}) \\&\quad = \frac{ (-1)^{a_D} t_{k+1}(\bar{l}_{k+1}, a_Y) + \Pr (Y_{k+1}=1 \mid C_{k+1}=D_{k+1}= Y_{k}=0, \bar{L}_{k,i}, A = a_Y; \hat{\beta }_{Y}) }{\Pr (Y_{k+1}=1 \mid C_{j+1}=D_{k+1}= Y_{k}=0, \bar{L}_{k,i}, A = a_D; \hat{\beta }_{Y}) } \\&\qquad \times \frac{\prod _{j=0}^{k-1} (-1)^{a_D} t_{j+1}(\bar{l}_{j+1}, a_Y) + \Pr (Y_{j+1}=0 \mid C_{j+1}=D_{j+1}= Y_{j}=0, \bar{L}_{j,i}, A = a_Y; \hat{\beta }_{Y}) }{ \prod _{j=0}^{k-1} \Pr (Y_{j+1}=0 \mid C_{j+1}=D_{j+1}= Y_{j}=0, \bar{L}_{j,i}, A = a_D; \hat{\beta }_{Y}) }, \end{aligned}$$

see Appendix F for proof.

A formal sensitivity analysis can be conducted by repeatedly estimating $\hat{\nu }^{\dagger }_{2,k}$ for each choice of $t_k(\bar{l}_k, a_Y)$ for a set of functions $\mathcal {T} = \{ t_{k,\lambda }(\bar{l}_k, a_Y) : \lambda \}$, where $\lambda$ is a finite dimensional parameter and $t_{k,0}(\bar{l}_k, a_Y) \equiv 0$ describes the setting with no bias, that is, no unmeasured common causes of $Y_k$ and $D_j$ or of $Y_k$ and $L_j$, for any *j*, *k* such that $0 < j \le k$.

Subject matter knowledge can help us to reason about the sensitivity function $t_k(\bar{l}_k, a_Y)$. To fix ideas, suppose that the graph in Fig. 7f represents the blood pressure example, where $U_{L,Y}$ is an unmeasured common cause that increases the blood pressure ($L_k$) and the risk of kidney failure ($Y_k$). Then we would expect $t_k(\bar{l}_k, a_Y)$ to be negative due to selection over time; subjects who do not receive the treatment component that intensively reduces blood pressure ($a_D = 0$) are less likely to be alive with larger values of $U_{L,Y}$ compared to those who received the component that intensively reduces blood pressure ($a_D = 1$).

Our sensitivity analysis technique is inspired by Tchetgen Tchetgen (2014). However, unlike Tchetgen Tchetgen (2014), the terms in our sensitivity function are not cross-world quantities that are unobservable in principle, but conditional expectations that can be identified in a future experiment in which $A_Y$ and $A_D$ are randomly assigned.

Furthermore, note that our identification results from Sect. 6 also motivate sensitivity analyses of violations of the isolation conditions from Sect. 5. In particular, suppose that an investigator assumed that full isolation was satisfied and, thus, used the simplified identification formula that was given in Stensrud et al. (2020). Then, the assumption of full isolation could be falsified by comparing these estimates to estimates derived from the estimators in Sect. 7, only assuming $Z_k$ partition. To do this sensitivity analysis, the investigator needs to measure a set of time-varying covariates $L_k, k \in \{0,\dots , K\}$.

## Discussion

We have provided generalized results for interpretation and identification of separable effects in competing events settings. These results allow the separable effects to be identified and meaningfullly interpreted in much broader settings than those initially considered by Stensrud et al. (2020). Generally these effects clarify the interpretation of total effects when competing events are affected by treatment, provide more information to patients and doctors for current treatment decisions and inform the development of improved treatments with unwanted components removed. In general, our framework provides a basis that allows subject matter experts to formally reason about the mechanisms by which treatments act on time-to-event outcomes and subsequently falsify this reasoning in a future trial.

Even under our generalized conditions, the separable effects may be difficult to identify given currently available data in many studies. However, they can point to shortcomings of the data typically collected in studies of competing events, and may guide the planning for improved data collection in future studies. This is particularly true of randomized trials which have historically relied heavily on the treatment randomization; failing to collect data on baseline and time-varying covariates makes it nearly impossible to adjust for selection bias due to censoring and/or to target estimands other than the total effect of the randomization. Furthermore, we outlined strategies for sensitivity analysis to both the dismissible component conditions and isolation conditions in Appendix F.

We have focused on establishing fundamental results for interpretation and identification of separable effects, as well as suggesting three estimators that are easy to implement. In future work, we aim to derive new estimators from the efficient influence function, which may achieve parametric convergence rates even when machine learning methods are used for model fitting (Robins et al. 1994; Chernozhukov et al. 2018; Robins et al. 2017; Van der Laan and Rose 2018), such that e.g. bias-aware model selection can be performed to minimize bias due to model misspecification (Cui and Tchetgen Tchetgen 2019). We will also extend our results to separable effects of time-varying treatment interventions (Robins et al. 2020) in competing events settings, including per-protocol effects in trials with nonadherence.

### Supplementary Information

Below is the link to the electronic supplementary material.

Supplementary material 1 (r 19 KB)

Supplementary material 2 (pdf 194 KB)

## Figures and Tables

**Fig. 10:**
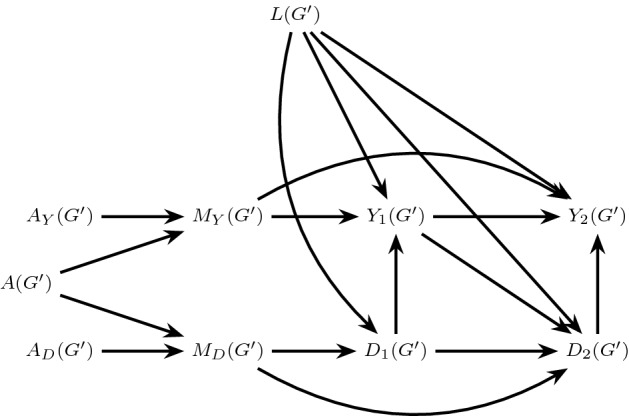
Modified DAG including the additional variables $M_{Y}$ and $M_{D}$ and their relation to *A*, $A_{Y}$ and $A_{D}$

**Fig. 11:**
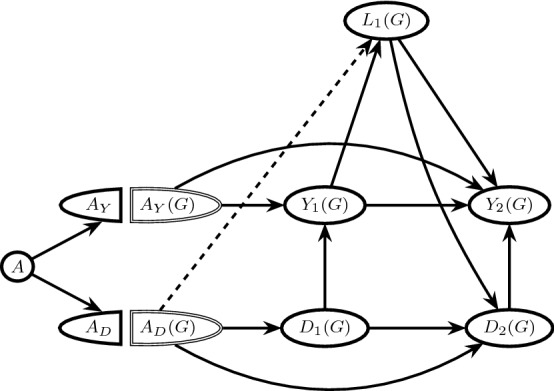
Single World Intervention Graph (SWIG) (Richardson and Robins 2013)

## Appendices

- Appendix A: Modified treatment assumption.
- Appendix B: Proof of identifiability.
- Appendix C $Z_k$ partition and the dismissible component conditions.
- Appendix D Proof of weighted representation of (20).
- Appendix E Estimation algorithms.
- Appendix F Proof of sensitivity analysis.

## A Modified treatment assumption

To define the generalized decomposition assumption in Sect. 4, we considered a decomposition of treatment *A* into different components, $A_Y$ and $A_D$, satisfying (2). Yet, a physical decomposition of *A* into components $A_Y$ and $A_D$ is not necessary for the validity of our results on identification and estimation of separable effects in Sects. 6–7. Specifically, the proofs in Appendix B only require condition (3) of the generalized decomposition assumption, which may also hold for treatments $A_Y$ and $A_D$ that are not components of *A*.

In this case, the separable effects can still be meaningfully interpreted as the effects of joint assignment to alternative treatments $A_Y$ and $A_D$ in place of assignment to *A*. The isolation conditions of Sect. 5 still constitute additional mechanistic assumptions on how these alternative treatments operate on the event of interest and the competing event. In the main text we defined a *G* transformation as a transformation of an extended causal DAG under the assumption of a physical decomposition. More generally, a *G* transformation can be understood as a (conventional) causal DAG representing data generating assumptions in a trial in which $A_Y$ and $A_D$ are randomly assigned. Thus, the *G* transformation graph differs from the causal DAG describing the observed data in that it depicts assumptions about a study in which the alternative treatments $A_Y$ and $A_D$, rather than the original study treatment *A*, are assigned, including how these treatments operate on future events. The isolation conditions, which only refer to assumptions about the mechanisms by which the alternative treatments $A_Y$ and $A_D$ operate, can thus be evaluated in *G* transformation graphs, even when $A_Y$ and $A_D$ are not assumed a physical decomposition of *A*. The *G* transformations are isomorphic to dynamic Single World Intervention Graphs (SWIGs) (Richardson and Robins 2013).

However, when treatments $A_Y$ and $A_D$ are not components of *A*, we require additional assumptions beyond (3) for the separable effects to *explain* the mechanism by which the original treatment *A* exerts its effects on $Y_{k+1}$ for $k \in \{0, \dots , K \}$. The following is an alternative assumption to (2) that, when coupled with (3), is sufficient for the separable effects to explain the total effect of the original treatment *A* on the event of interest when $A_Y$ and $A_D$ are not a decomposition of *A*. For variables, $M_Y$ and $M_D$, consider the following assumption:

27 $$\begin{aligned}&A_Y\text { and }A_D\text { exert all their effects through }M_Y\text { and } M_D,\text { respectively, and} \nonumber \\&\quad M^{a_Y=a,a_D}_Y = M^{a}_Y \quad \text { for } a_D \in \{0,1\}, \quad M^{a_Y,a_D=a}_D = M^{a}_D \quad \text { for } a_Y \in \{0,1\} , \end{aligned}$$

where the counterfactuals on the right and left hand side of both equalities in (27) refer to assignment to $A=a$ and no level of $A_Y$ and $A_D$, and assignment to ($A_Y=a_Y$, $A_D=a_D$) and no level of *A*, respectively. A transformed DAG that is consistent with (27) is shown in Fig. 10 where $G'$ refers to a six arm trial in which subjects are either randomly assigned to *A* (and no level of $A_Y$ and $A_D$) or a combination of $A_Y=a_Y$ and $A_D=a_D$ (and no level of *A*).

Note that assumptions (3) and (27) can, in principle, be falsified in a future randomized experiment. For example, by randomly assigning individuals to *A* or a joint treatment $(A_Y,A_D)$, we can assess whether $E(W \mid A_Y=a, A_D=a)\ne E(W \mid A=a)$, for any $W \in \{Y_{1},\dots ,Y_{K+1},D_{1},\dots ,D_{k+1},Z_{1},\dots ,Z_{k+1}, M_Y, M_D\}$.

To fix ideas, consider a study of estrogen therapy versus placebo in men with prostate cancer, which was the running example in Stensrud et al. (2020). Estrogen therapy is thought to reduce death due to prostate cancer, because it reduces testosterone levels and thus prevents the cancer cells from growing. However, there is concern that estrogen therapy may also increase mortality due to cardiovascular disease, e.g. through estrogen-induced synthesis of coagulation factors (Turo et al. 2014). Stensrud et al. (2020) used this example to motivate the separable direct and indirect effects under full isolation, and suggested that alternative treatments, such as castration and luteinizing hormone releasing hormone (LHRH) antagonists, can have the same effect as estrogen on testosterone reduction ($M_Y$), but, unlike estrogen, these treatments do not exert effects on the coagulation factors ($M_D$). Whereas Stensrud et al. (2020) did not formally define the variables $M_Y$ and $M_D$, providing the story that includes these additional variables, satisfying (27), is essential to connect the effect of e.g. $A_Y=1$ (here, assigning testosterone or LHRH antagonists) to the separable direct and indirect effects of estrogen therapy (*A*) itself.

## B Proof of identifiability

Before we provide a proof of identification formula (20), consider the following identifiability conditions that generalize the conditions from Sect. 6 to allow for censoring.

1. Exchangeability:
  28
  29
  Condition (28) holds when $A_Y$ and $A_D$ are randomly assigned at baseline, possibly conditional on $L_0$. Condition (29) requires that losses to follow-up are independent of future counterfactual events, given the measured past; this assumption does not hold by design in a randomised trial, as losses to follow-up are not randomly assigned in practice.
2. Positivity:
  30 $$\begin{aligned}&f(L_0=l_0)>0\implies \nonumber \\&\quad \Pr (A=a\mid L_0=l_0)>0, \end{aligned}$$
  31 $$\begin{aligned}&f_{\overline{L}_k,D_{k+1},C_{k+1},Y_k}(\overline{l}_k,0,0,0)> 0 \implies \nonumber \\&\quad \Pr (A=a|D_{k+1}=C_{k+1}=Y_k=0,\overline{L}_k=\overline{l}_k)>0 \end{aligned}$$
  32 $$\begin{aligned}&\Pr (A = a,Y_k=0,D_k=0,\bar{C}_k=0,\bar{L}_k=\overline{l}_k)> 0 \implies \nonumber \\&\quad \Pr (C_{k+1}=0\mid Y_k=0,D_k=0,\bar{C}_k=0,\bar{L}_k=\overline{l}_k,A=a) > 0 \end{aligned}$$
  for $a\in \{0,1\}$, $k \in \{0, \dots ,K\}$ and $L_k \in \mathcal {L}$. Conditions (30) and (31) were described in the main text. Condition (32) requires that for any possible history of treatment assignment and covariates among those who are event-free and uncensored at *k*, some subjects will remain uncensored at $k+1$.
3. Consistency:
  33 $$\begin{aligned}&\text {if } A=a \text { and } \bar{C}_{k+1} = 0, \nonumber \\&\text {then } \bar{Y}_{k+1} = \bar{Y}^{a, \bar{c}=0}_{k+1}, \bar{D}_{k+1} = \bar{D}^{a, \bar{c}=0}_{k+1} \text { and } \bar{L}_{k+1} = \bar{L}^{a,\bar{c}=0}_{k+1}. \end{aligned}$$
  Consistency is satisfied if any individual who has data history consistent with the intervention under a counterfactual scenario, would have an observed outcome that is equal to the counterfactual outcome.
4. Dismissible component conditions:
  34
  35
  36
  37
  The dismissible component conditions are identical to the conditions in Sect. 6, but the superscript ${\bar{c}=0}$ is included to emphasize that we consider outcomes in a setting in which loss to follow-up is eliminated even under *G*.

### Lemma 1

Under a FFRCISTG model, the dismissible component conditions (34)–(37) imply the following equalities for $a_Y,a_D \in \{0,1\}$:

38 $$\begin{aligned}&\Pr (Y^{a_Y,a_D=0,\bar{c}= 0}_{k+1} = 1 \mid Y^{a_Y,a_D=0,\bar{c}= 0}_k = 0, D^{a_Y,a_D=0,\bar{c}= 0}_{k+1} = 0,\bar{L}^{a_Y,a_D=0,\bar{c}= 0}_{k} = \bar{l}_{k}) \end{aligned}$$

39 $$\begin{aligned}&\quad =\Pr (Y^{a_Y,a_D=1,\bar{c}= 0}_{k+1} = 1 \mid Y^{a_Y,a_D=1,\bar{c}=0}_k = D^{a_Y,a_D=1,\bar{c}=0}_{k+1} = 0 , \bar{L}^{a_Y,a_D=1,\bar{c}= 0}_{k} = \bar{l}_{k} ), \nonumber \\&\Pr (D^{a_Y=0,a_D,\bar{c}= 0}_{k+1} = 1 \mid Y^{a_Y=0,a_D,\bar{c}= 0}_k = D^{a_Y=0,a_D,\bar{c}= 0}_k = 0, \bar{L}^{a_Y=0,a_D,\bar{c}= 0}_{k} = \bar{l}_{k}) \end{aligned}$$

40 $$\begin{aligned}&\quad \!=\!\Pr (D^{a_Y=1,a_D,\bar{c}= 0}_{k+1} \!=\! 1 \mid Y^{a_Y=1,a_D,\bar{c}=0}_k \!=\! 0, D^{a_Y=1,a_D,\bar{c}=0}_k \!=\! 0 , \bar{L}^{a_Y=1,a_D,\bar{c}= 0}_{k} = \bar{l}_{k} ), \nonumber \\&f(L^{a_Y,a_D=1,\bar{c}= 0}_{Y,k+1} = l_{Y,k+1} \mid Y^{a_Y,a_D=1,\bar{c}= 0}_{k+1} = D^{a_Y,a_D=1,\bar{c}= 0}_{k+1} = 0, \end{aligned}$$

41 $$\begin{aligned}&\quad \bar{L}^{a_Y,a_D=1,\bar{c}= 0}_{k} = \bar{l}_{k},L^{a_Y,a_D=1,\bar{c}= 0}_{D,k+1} = l_{D,k+1}) \nonumber \\&\quad =\Pr (L^{a_Y,a_D=0,\bar{c}= 0}_{Y,k+1} = l_{Y,k+1} \mid Y^{a_Y,a_D=0,\bar{c}= 0}_{k+1} = D^{a_Y,a_D=0,\bar{c}= 0}_{k+1} = 0, \nonumber \\&\quad \bar{L}^{a_Y,a_D=0,\bar{c}= 0}_{k} = \bar{l}_{k},L^{a_Y,a_D=0,\bar{c}= 0}_{D,k+1} = l_{D,k+1}), \nonumber \\&f(L^{a_Y=0,a_D,\bar{c}= 0}_{D,k+1} = l_{D,k+1} \mid Y^{a_Y=0,a_D,\bar{c}= 0}_{k+1} = D^{a_Y=0,a_D,\bar{c}= 0}_{k+1} = 0, \bar{L}^{a_Y=0,a_D,\bar{c}= 0}_{k} = \bar{l}_{k}) \end{aligned}$$

42 $$\begin{aligned}&\quad =f(L^{a_Y=1,a_D,\bar{c}= 0}_{D,k+1} = l_{D,k+1} \mid Y^{a_Y=1,a_D,\bar{c}= 0}_{k+1} = D^{a_Y=1,a_D,\bar{c}= 0}_{k+1} = 0,\nonumber \\&\quad \bar{L}^{a_Y=1,a_D,\bar{c}= 0}_{k} = \bar{l}_{k}). \nonumber \end{aligned}$$

### Proof

$$\begin{aligned}&\Pr (Y^{a_Y,a_D=0,\bar{c}= 0}_{k+1} = 1 \mid Y^{a_Y,a_D=0,\bar{c}= 0}_k = 0, D^{a_Y,a_D=0,\bar{c}= 0}_{k+1} = 0,\bar{L}^{a_Y,a_D=0,\bar{c}= 0}_{k} = \bar{l}_{k} ) \\&\quad = \Pr (Y^{\bar{c}= 0}_{k+1}(G)=1 \mid Y^{\bar{c}= 0}_{k}(G)=0,D^{\bar{c}= 0}_{k+1}(G)=0, \bar{L}^{\bar{c}= 0}_{k}(G)= \bar{l}_{k},\\&\quad A_Y(G)=a_Y,A_D(G)=0) \text { by def. of } G \\&\quad = \Pr (Y^{\bar{c}= 0}_{k+1}(G)=1 \mid Y^{\bar{c}= 0}_{k}(G)=0,D^{\bar{c}= 0}_{k+1}(G)=0, \bar{L}^{\bar{c}= 0}_{k}(G)= \bar{l}_{k},\\&\quad A_Y(G)=a_Y,A_D(G)=1) \text { due to }(34) \\&\quad = \Pr (Y^{a_Y,a_D=1,\bar{c}= 0}_{k+1} = 1 \mid Y^{a_Y,a_D=1,\bar{c}= 0}_k = 0, D^{a_Y,a_D=1,\bar{c}= 0}_{k+1} = 0,\\&\quad \bar{L}^{a_Y,a_D=1,\bar{c}= 0}_{k} = \bar{l}_{k} ) \text { by def. of } G, \\ \end{aligned}$$

which shows that equality (38) holds, and (39)–(41) can be shown from analogous arguments, where we use conditions (35)–(37) in the second step, respectively, instead of (34). $\square$

### Lemma 2

Suppose that conditions (28)–(33) hold. Then, for $s = 0,\dots , K$ and $a_Y,a_D \in \{0,1\}$,

43 $$\begin{aligned} \Pr&(Y^{a_Y=a_D=a, \bar{c}=0}_{s+1}=1 \mid D^{a_Y=a_D=a, \bar{c}=0}_{s+1}= Y^{a_Y=a_D=a, \bar{c}=0}_{s}=0,\bar{L}^{a_Y=a_D=a, \bar{c}=0}_{s} = \bar{l}_{s}) \nonumber \\&\quad =\Pr (Y_{s+1}{=}1 \mid C_{s+1}{=} D_{s+1}= Y_{s}=0,\bar{L}_{s} {=} \bar{l}_{s}, A = a), \end{aligned}$$

44 $$\begin{aligned} \Pr&(D^{a_Y=a_D=a, \bar{c}=0}_{s+1}=1 \mid D^{a_Y=a_D=a, \bar{c}=0}_{s}= Y^{a_Y=a_D=a, \bar{c}=0}_{s}=0,\bar{L}^{a_Y=a_D=a, \bar{c}=0}_{s} = \bar{l}_{s}) \nonumber \\&\quad = \Pr (D_{s+1}=1 \mid C_{s+1} = D_{s}= Y_{s}=0,\bar{L}_{s} = \bar{l}_{s}, A=a), \end{aligned}$$

45 $$\begin{aligned} \Pr (&L^{a_Y=a_D=a, \bar{c}=0}_{Y, s} {=} l_{Y,s} \mid Y^{a_Y=a_D=a, \bar{c}=0}_{s} {=} D^{a_Y=a_D=a, \bar{c}=0}_{s} = 0, \bar{L}^{a_Y=a_D=a, \bar{c}=0}_{s-1} {=} \bar{l}_{s-1},\nonumber \\&\quad L_{D,s}^{a_Y=a_D=a, \overline{c}=0} = l_{D,s}) \nonumber \\&\quad =f(L_{Y,s} = \bar{l}_{Y,s} \mid C_{s} = D_{s} = Y_{s} = 0, \bar{L}_{s-1}=\bar{l}_{s-1}, A=a, L_{D,s} = l_{D,s}), \end{aligned}$$

46 $$\begin{aligned} \Pr (&L^{a_Y=a_D=a, \bar{c}=0}_{D, s} {=} l_{D,s} \mid Y^{a_Y=a_D=a, \bar{c}=0}_{s} {=} D^{a_Y=a_D=a, \bar{c}=0}_{s} {=} 0, \bar{L}^{a_Y=a_D=a, \bar{c}=0}_{s-1} = \bar{l}_{s-1}) \nonumber \\&= f(L_{D,s} = l_{D,s} \mid C_{s} = D_{s} = Y_{s} = 0, \bar{L}_{s-1}=\bar{l}_{s-1}, A = a) . \end{aligned}$$

### Proof

Consider first (42),

$$\begin{aligned}&\Pr (Y^{a_Y=a_D=a, \bar{c}=0}_{s+1}=1 \mid D^{a_Y=a_D=a, \bar{c}=0}_{s+1}= Y^{a_Y=a_D=a, \bar{c}=0}_{s}=0,\bar{L}^{a_Y=a_D=a, \bar{c}=0}_{s} = \bar{l}_{s}) \\&\quad = \Pr (Y^{a, \bar{c}=0}_{s+1}=1 \mid D^{a, \bar{c}=0}_{s+1}= Y^{a, \bar{c}=0}_{s}=0,\bar{L}^{a, \bar{c}=0}_{s} = \bar{l}_{s}) \\&\quad = \Pr (Y^{a, \bar{c}=0}_{s+1}=1 \mid D^{a, \bar{c}=0}_{s+1}= Y^{a, \bar{c}=0}_{s}=Y_{0}=D_{0}=\bar{C}_{0}=0,L_0=l_0,\bar{L}^{a, \bar{c}=0}_{s} = \bar{l}_{s}) \\&\quad = \frac{\Pr (Y^{a, \bar{c}=0}_{s+1}=1, \bar{D}^{a, \bar{c}=0}_{s+1}= \bar{Y}^{a, \bar{c}=0}_{s}=0, \bar{L}^{a, \bar{c}=0}_{s} = \bar{l}_{s} \mid Y_{0}=D_{0}=\bar{C}_{0}=0,L_0=l_{0}, A=a)}{\Pr (\bar{D}^{a, \bar{c}=0}_{s+1}= \bar{Y}^{a, \bar{c}=0}_{s}=0, \bar{L}^{a, \bar{c}=0}_{s} = \bar{l}_{s} \mid Y_{0}=D_{0}=\bar{C}_{0}=0,L_{0}=l_{0}, A=a)}, \\ \end{aligned}$$

where we used the fact that all subjects are event-free and uncensored at $s=0$, laws of probability and (28). Using (29) and positivity,

47 $$\begin{aligned}&\frac{\Pr (Y^{a, \bar{c}=0}_{s+1}=1, \bar{D}^{a, \bar{c}=0}_{s+1}= \bar{Y}^{a, \bar{c}=0}_{s}=0, \bar{L}^{a, \bar{c}=0}_{s} = \bar{l}_{s} \mid Y_{0}=D_{0}=\bar{C}_{1}=0,L_0=l_0, A=a)}{\Pr (\bar{D}^{a, \bar{c}=0}_{s+1}= \bar{Y}^{a, \bar{c}=0}_{s}=0, \bar{L}^{a, \bar{c}=0}_{s} = \bar{l}_{s} \mid Y_{0}=D_{0}=\bar{C}_{1}=0,L_0=l_0, A=a)}, \nonumber \\&\quad = \Pr (Y^{a, \bar{c}=0}_{s+1}=1 \mid D^{a, \bar{c}=0}_{s+1}= Y^{a, \bar{c}=0}_{s}=0,\bar{L}^{a, \bar{c}=0}_{s} {=} \bar{l}_{s}, Y_{0}=D_{0}{=}\bar{C}_{1}=0,L_0{=}l_0, A=a).\nonumber \\ \end{aligned}$$

For $s=0$, under consistency,

48 $$\begin{aligned}&\Pr (Y^{a, \bar{c}=0}_{s+1}=1 \mid D^{a, \bar{c}=0}_{s+1}= Y^{a, \bar{c}=0}_{s}=0,\bar{L}^{a, \bar{c}=0}_{s} = \bar{l}_{s}, Y_{0}=D_{0}=\bar{C}_{1}=0,L_0=l_0, A=a) \nonumber \\&\quad = \Pr (Y^{a, \bar{c}=0}_{s+1}=1 \mid D^{a, \bar{c}=0}_{s+1}= Y^{a, \bar{c}=0}_{s}=0,\bar{L}^{a, \bar{c}=0}_{s} {=} \bar{l}_{s}, Y_{0}=D_{1}=\bar{C}_{1}{=}0,{L}_0= {l}_{0}, A=a) \nonumber \\&\quad = \Pr (Y^{a, \bar{c}=0}_{s+1}=1 \mid Y_{0}=D_{1}=\bar{C}_{1}=0,{L}_0=l_{0}, A=a) \end{aligned}$$

which proves the lemma for $s=0$.

Further, for $s>1$, using consistency,

49 $$\begin{aligned}&\Pr (Y^{a, \bar{c}=0}_{s+1}=1 \mid D^{a, \bar{c}=0}_{s+1}= Y^{a, \bar{c}=0}_{s}=0,\bar{L}^{a, \bar{c}=0}_{s} = \bar{l}_{s}, Y_{0}=D_{0}=\bar{C}_{1}=0,L_0=l_0, A=a) \nonumber \\&\quad = \Pr (Y^{a, \bar{c}=0}_{s+1}=1 \mid D^{a, \bar{c}=0}_{s+1}= Y^{a, \bar{c}=0}_{s}{=}0,\bar{L}^{a, \bar{c}=0}_{s} {=} \bar{l}_{s}, Y_{1}=D_{1}=\bar{C}_{1}{=}0,\overline{L}_1=\overline{l}_{1}, A=a)\nonumber \\ \end{aligned}$$

Apply (29) and positivity again,

50 $$\begin{aligned}&\Pr (Y^{a, \bar{c}=0}_{s+1}=1 \mid D^{a, \bar{c}=0}_{s+1}= Y^{a, \bar{c}=0}_{s}=0,\bar{L}^{a, \bar{c}=0}_{s} = \bar{l}_{s}, Y_{1}=D_{1}=\bar{C}_{1}=0,\overline{L}_1=\overline{l}_{1}, A=a) \nonumber \\&\quad = \frac{\Pr (Y^{a, \bar{c}=0}_{s+1}=1, \bar{D}^{a, \bar{c}=0}_{s+1}{=} \bar{Y}^{a, \bar{c}=0}_{s}=0, \bar{L}^{a, \bar{c}=0}_{s} {=} \bar{l}_{s} \mid Y_{1}=D_{1}=\bar{C}_{2}{=}0,\overline{L}_1=\overline{l}_{1}, A=a)}{\Pr (\bar{D}^{a, \bar{c}=0}_{s+1}= \bar{Y}^{a, \bar{c}=0}_{s}=0, \bar{L}^{a, \bar{c}=0}_{s} = \bar{l}_{s} \mid Y_{1}=D_{1}=\bar{C}_{2}=0,\overline{L}_1=\overline{l}_{1}, A=a)}, \nonumber \\&\quad = \Pr (Y^{a, \bar{c}=0}_{s+1}{=}1 \mid D^{a, \bar{c}=0}_{s+1}= Y^{a \bar{c}=0}_{s}=0,\bar{L}^{a, \bar{c}=0}_{s} {=} \bar{l}_{s}, Y_{1}=D_{1}=\bar{C}_{2}{=}0,\overline{L}_1=\overline{l}_{1}, A=a).\nonumber \\ \end{aligned}$$

Using consistency,

51 $$\begin{aligned}&\Pr (Y^{a, \bar{c}=0}_{s+1}=1 \mid D^{a, \bar{c}=0}_{s+1}= Y^{a, \bar{c}=0}_{s}=0,\bar{L}^{a, \bar{c}=0}_{s} = \bar{l}_{s}, Y_{1}=D_{1}=\bar{C}_{2}=0,\overline{L}_1=\overline{l}_{1}, A=a) \nonumber \\&\quad = \Pr (Y^{a, \bar{c}=0}_{s+1}=1 \mid D^{a, \bar{c}=0}_{s+1}= Y^{a, \bar{c}=0}_{s}=0,\bar{L}^{a, \bar{c}=0}_{s} = \bar{l}_{s}, Y_{2}=D_{2}=\bar{C}_{2}=0,\overline{L}_2=\overline{l}_{2}, A=a)\nonumber \\ \end{aligned}$$

Arguing iteratively, we find that

52 $$\begin{aligned}&\Pr (Y^{a, \bar{c}=0}_{s+1}=1 \mid D^{a, \bar{c}=0}_{s+1}= Y^{a, \bar{c}=0}_{s}=0,\bar{L}^{a, \bar{c}=0}_{s} = \bar{l}_{s}) \nonumber \\&\quad = \Pr (Y_{s+1}=1 \mid C_{s+1}= D_{s+1}= Y_{s}=0,\bar{L}_{s} = \bar{l}_{s}, A=a). \end{aligned}$$

Analogous arguments can be used to show (43)–(45). $\square$

### Theorem 1

Suppose conditions (28)–(37) hold. Then, for $a_Y,a_D \in \{0,1\}$,

$$\begin{aligned} \Pr (&Y_{k+1}^{a_Y,a_D,\bar{c}=0}=1) \\&\quad = \sum _{\bar{l}_K} \Big [ \sum _{s=0}^{K} \Pr (Y_{s+1}=1 \mid C_{s+1}= D_{s+1}= Y_{s}=0, \bar{L}_{s} = \bar{l}_{s}, A = a_Y) \nonumber \\&\prod _{j=0}^{s} \big \{ \Pr (D_{j+1}=0 \mid C_{j+1}=D_{j}= Y_{j}=0, \bar{L}_{j} = \bar{l}_{j}, A = a_D) \nonumber \\&\qquad \times \Pr (Y_{j}=0 \mid C_{j}=D_{j}= Y_{j-1}=0, \bar{L}_{j-1} = \bar{l}_{j-1}, A = a_Y) \nonumber \\&\qquad \times f(L_{Y,j} = l_{Y,j} \mid C_{j}= Y_{j} = D_{j} = 0, \bar{L}_{j-1} = \bar{l}_{j-1}, L_{D,j} = l_{D,j}, A = a_Y) \nonumber \\&\qquad \times f(L_{D,j} = l_{D,j} \mid C_{j}= Y_{j} = D_{j} = 0, \bar{L}_{j-1} = \bar{l}_{j-1}, A = a_D) \big \} \Big ]. \end{aligned}$$

### Proof

If $a_Y=a_D \in \{0,1\}$, it is straightforward to use laws of probability to show that the theorem holds. Consider now the case where $a_Y \ne a_D$. In particular, let $a_Y=1 \ne a_D=0$. Using laws of probability and Lemma 1,

53 $$\begin{aligned} \Pr&(Y^{a_Y=1,a_D=0, \bar{c}=0}_{K+1}=1) \nonumber \\&\quad = \sum _{\bar{l}_K} \Pr (Y^{a_Y=1,a_D=0, \bar{c}=0}_{K+1}=1 \mid \bar{L}^{a_Y=1,a_D=0, \bar{c}=0}_K = \bar{l}_K) \Pr (\bar{L}^{a_Y=1,a_D=0, \bar{c}=0}_K = \bar{l}_K) \nonumber \\&\quad = \sum _{\bar{l}_K} \Big [ \sum _{s=0}^{K} \Pr (Y^{a_Y=1,a_D=0, \bar{c}=0}_{s+1}=1 \mid D^{a_Y=1,a_D=0, \bar{c}=0}_{s+1}\nonumber \\&\quad = Y^{a_Y=1,a_D=0, \bar{c}=0}_{s}=0, \bar{L}^{a_Y=1,a_D=0, \bar{c}=0}_{s} = \bar{l}_{s}) \nonumber \\&\prod _{j=0}^{s} \big \{ \Pr (D^{a_Y=1,a_D=0, \bar{c}=0}_{j+1}=0 \mid D^{a_Y=1,a_D=0, \bar{c}=0}_{j}= Y^{a_Y=1,a_D=0, \bar{c}=0}_{j}=0,\nonumber \\&\quad \bar{L}^{a_Y=1,a_D=0, \bar{c}=0}_{j} = \bar{l}_{j}) \nonumber \\&\qquad \times \Pr (Y^{a_Y=1,a_D=0, \bar{c}=0}_{j}=0 \mid D^{a_Y=1,a_D=0, \bar{c}=0}_{j}= Y^{a_Y=1,a_D=0, \bar{c}=0}_{j-1}=0,\nonumber \\&\quad \bar{L}^{a_Y=1,a_D=0, \bar{c}=0}_{j} = \bar{l}_{j})\nonumber \\&\qquad \times f(L^{a_Y=1,a_D=0,\bar{c}= 0}_{Y,j} = l_{Y,j} \mid Y^{a_Y=1,a_D=0,\bar{c}= 0}_{j} = D^{a_Y=1,a_D=0,\bar{c}= 0}_{j} = 0\nonumber \\&\quad \bar{L}^{a_Y=1,a_D=0,\bar{c}= 0}_{j-1} = \bar{l}_{j-1}, L^{a_Y=1,a_D=0,\bar{c}= 0}_{D,j} = l_{D,j}) \nonumber \\&\qquad \times f(L^{a_Y=1,a_D=0,\bar{c}= 0}_{D, j} = l_{D,j} \mid Y^{a_Y=1,a_D=0,\bar{c}= 0}_{j} = D^{a_Y=1,a_D=0,\bar{c}= 0}_{j} = 0,\nonumber \\&\quad \bar{L}^{a_Y=1,a_D=0,\bar{c}= 0}_{j-1} = \bar{l}_{j-1}) \big \} \Big ] \nonumber \\&\quad = \sum _{\bar{l}_K} \Big [ \sum _{s=0}^{K} \Pr (Y^{a=1, \bar{c}=0}_{s+1}=1 \mid D^{a=1, \bar{c}=0}_{s+1}= Y^{a=1, \bar{c}=0}_{s}=0,\nonumber \\&\quad \bar{L}^{A_Y = A_D = a1, \bar{c}=0}_{s} =\overline{l}_s) \nonumber \\&\prod _{j=0}^{s} \big \{ \Pr (D^{a=0, \bar{c}=0}_{j+1}=0 \mid D^{a=0, \bar{c}=0}_{j}= Y^{a=0, \bar{c}=0}_{j}=0, \bar{L}^{a=0, \bar{c}=0}_{j} = \bar{l}_{j}) \nonumber \\&\qquad \times \Pr (Y^{a=1, \bar{c}=0}_{j}=0 \mid D^{a=1, \bar{c}=0}_{j}= Y^{a=1, \bar{c}=0}_{j-1}=0, \bar{L}^{a=1, \bar{c}=0}_{j}= \bar{l}_{j}) \nonumber \\&\qquad \times f(L^{a=1, \bar{c}=0}_{Y,j} = l_{Y,j} \mid Y^{a=1, \bar{c}=0}_{j} = D^{a=1, \bar{c}=0}_{j} = 0, \bar{L}^{a=1, \bar{c}=0}_{j-1} = \bar{l}_{j-1},\nonumber \\&\quad L^{a=1,\bar{c}= 0}_{D,j} = l_{D,j}) \nonumber \\&\qquad \times f(L^{a=0, \bar{c}=0}_{D,j} = l_{D,j} \mid Y^{a=0, \bar{c}=0}_{j} = D^{a=0, \bar{c}=0}_{j} = 0, \bar{L}^{a=0, \bar{c}=0}_{j-1} = \bar{l}_{j-1}) \big \} \Big ], \end{aligned}$$

where $Y^{a_Y,a_D, \bar{c}=0}_{-1}$, and $L^{a_Y,a_D, \bar{c}=0}_{-1}$ are empty sets. Using Lemma 2, we can substitute the terms in the last equality in (52),

$$\begin{aligned}&= \sum _{\bar{l}_K} \Big [ \sum _{s=0}^{K} \Pr (Y^{a=1, \bar{c}=0}_{s+1}=1 \mid D^{a=1, \bar{c}=0}_{s+1}= Y^{a=1, \bar{c}=0}_{s}=0, \bar{L}^{A_Y = A_D = a_Y, \bar{c}=0}_{s} = \overline{l}_s ) \\&\quad \prod _{j=0}^{s} \big \{ \Pr (D^{a=0, \bar{c}=0}_{j+1}=0 \mid D^{a=0, \bar{c}=0}_{j}= Y^{a=0, \bar{c}=0}_{j}=0, \bar{L}^{a=0, \bar{c}=0}_{j} = \bar{l}_{j}) \\&\qquad \times \Pr (Y^{a=1, \bar{c}=0}_{j}=0 \mid D^{a=1, \bar{c}=0}_{j}= Y^{a=1, \bar{c}=0}_{j-1}=0, \bar{L}^{a=1, \bar{c}=0}_{j-1}= \bar{l}_{j-1}) \\&\qquad \times f(L^{a=1, \bar{c}=0}_{Y,j} = l_{Y,j} \mid Y^{a=1, \bar{c}=0}_{j} = D^{a=1, \bar{c}=0}_{j} = 0, \bar{L}^{a=1, \bar{c}=0}_{j-1} = \bar{l}_{j-1}, L^{a=1,\bar{c}= 0}_{D,j} = l_{D,j}) \\&\qquad \times f(L^{a=0, \bar{c}=0}_{D,j} = l_{D,j} \mid Y^{a=0, \bar{c}=0}_{j} = D^{a=0, \bar{c}=0}_{j} = 0, \bar{L}^{a=0, \bar{c}=0}_{j-1} = \bar{l}_{j-1}) \big \} \Big ] \\&\quad = \sum _{\bar{l}_K} \Big [ \sum _{s=0}^{K} \Pr (Y_{s+1}=1 \mid C_{s+1}= D_{s+1}= Y_{s}=0, \bar{L}_{s} = \bar{l}_{s}, A = a_Y) \\&\quad \prod _{j=0}^{s} \big \{ \Pr (D_{j+1}=0 \mid C_{j+1}=D_{j}= Y_{j}=0, \bar{L}_{j} = \bar{l}_{j}, A = a_D) \\&\qquad \times \Pr (Y_{j}=0 \mid C_{j}=D_{j}= Y_{j-1}=0, \bar{L}_{j-1} = \bar{l}_{j-1}, A = a_Y) \\&\qquad \times f(L_{Y,j} = l_{Y,j} \mid C_{j}= Y_{j} = D_{j} = 0, \bar{L}_{j-1} = \bar{l}_{j-1}, L_{D,j} = l_{D,j}, A = a_Y) \\&\qquad \times f(L_{D,j} = l_{D,j} \mid C_{j}= Y_{j} = D_{j} = 0, \bar{L}_{j-1} = \bar{l}_{j-1}, A = a_D) \big \} \Big ]. \end{aligned}$$

$$\square $$

## C $Z_k$ partition and the dismissible component conditions

### Lemma 3

$Z_k$ partition fails if and only if any of the following statements are true for some $k \in \{1,\dots , K\}$: (i) there is a direct arrow from $A_Y$ into $D_{k+1}$, (ii) there is a direct arrow from $A_D$ into $Y_{k+1}$, or (iii) there exists a node $W \in \bar{Z}_k$ such that there are direct arrows from both $A_Y$ and $A_D$ into *W*.

### Proof

First we directly show that if (i), (ii) or (iii) holds then $Z_k$ partition fails. If (i) holds then (8) is violated by the presence of a causal path $A_Y\rightarrow D_{k+1}$ and therefore $Z_k$ partition is violated. If (ii) holds then (9) is violated by the presence of a causal path $A_D\rightarrow Y_{k+1}$ and therefore $Z_k$ partition is violated. Now suppose (iii) is true and define a partition of $Z_k$ such that $W \in \bar{Z}_{D,k}$. Then a causal path $A_Y\rightarrow W$ will exist and (8), and therefore $Z_k$ partition, is violated. Alternatively suppose (iii) is true and define a partition of $Z_k$ such that, instead, $W \in \bar{Z}_{Y,k}$ Then a causal path $A_D\rightarrow W$ will exist and (9), and therefore $Z_k$ partition, is violated.

Next we show by contradiction that if $Z_k$ partition fails then (i), (ii) or (iii) must hold. Suppose $Z_k$ partition holds and (i), (ii) or (iii) also hold. If (i), (ii) or (iii) hold then one of the following causal paths must be present: $A_Y\rightarrow D_{k+1}$, for some $k \in \{1, \dots ,K\}$; $A_D\rightarrow Y_{k+1}$, for some $k \in \{1, \dots ,K\}$; $A_Y\rightarrow W$ for some $W \in \bar{Z}_{D,K}$; or $A_D\rightarrow W$ for some $W \in \bar{Z}_{Y,K}$. The presence of any of these paths violates either (8) or (9) such that $Z_k$ partition fails. Thus, we have a contradiction and we are done. $\square$

### Lemma 4

If $Z_k$ partition fails, then at least one of the dismissible component conditions fail.

### Proof

Suppose $Z_k$ partition fails. Then, by lemma 3, (i), (ii) or (iii) must hold such that at least one of the following paths must be present for $W \in \bar{Z}_{j}$, $j \le k$, a cause of $Y_{k+1}$ and/or $D_{k+1}$, for some $k \in \{1, \dots ,K\}$: $A_Y \rightarrow D_{k+1}$; $A_D\rightarrow Y_{k+1}$; $A_Y \rightarrow W\rightarrow \ldots \rightarrow D_{k+1}$ and $A_D\rightarrow W \rightarrow \ldots \rightarrow D_{k+1}$; or $A_D\rightarrow W\rightarrow \ldots \rightarrow Y_{k+1}$ and $A_Y\rightarrow W\rightarrow \ldots \rightarrow Y_{k+1}$.

If the path $A_Y \rightarrow D_{k+1}$ is present then the dismissible component condition(15) fails for any choice of $\overline{L}_k$. If the path $A_D \rightarrow Y_{k+1}$ is present then the dismissible component condition (14) fails for any choice of $\overline{L}_k$.

Suppose $W \notin \bar{L}_K$, (*W* is unmeasured). If the path $A_Y\rightarrow W\rightarrow \ldots \rightarrow D_{k+1}$ is present then the dismissible component condition (15) fails for any choice of $\overline{L}_k$. If the path $A_D\rightarrow W\rightarrow Y_{k+1}$ is present then the dismissible component condition (14) fails for any choice of $\overline{L}_k$.

Suppose $W \in \bar{L}_{j+1}$ for some $j \in \{0, \dots ,K\}$ (*W* is measured). If the paths $A_Y\rightarrow W\rightarrow \ldots \rightarrow D_{k+1}$ and $A_D\rightarrow W\rightarrow \ldots \rightarrow D_{k+1}$ are present then, no matter our choice of $\overline{L}_j$, if we choose $W \in \bar{L}_{Y,j+1}$ the dismissible component condition (16) fails and if we choose $W \in \bar{L}_{D,j+1}$ the dismissible component condition (17) fails. Similarly, if the paths $A_D\rightarrow W\rightarrow \ldots \rightarrow Y_{k+1}$ and $A_Y\rightarrow W\rightarrow \ldots \rightarrow Y_{k+1}$ are present then, no matter our choice of $\overline{L}_j$, if we choose $W \in \bar{L}_{Y,j+1}$ the dismissible component condition (16) fails and if we choose $W \in \bar{L}_{D,j+1}$ the dismissible component condition (17) fails. $\square$

### Lemma 5

If the dismissible component conditions hold when we define $\overline{L}_{Y,K}=\emptyset$, then $A_Y$ partial isolation holds.

### Proof

We give a proof by contradiction. Suppose the dismissible component conditions hold under $L_{Y,k}=\emptyset$, but $A_Y$ partial isolation does not hold. Then, if there is a direct arrow $A_Y \rightarrow D_{k+1}$ for any $k \in \{0, \dots ,K\}$, this arrow would violate (15), which is a contradiction. Alternatively, $A_Y$ partial isolation can only be violated if there exists a *W* such that $A_Y \rightarrow W \rightarrow ... \rightarrow D_{k+1}$ for any $k \in \{0, \dots ,K\}$. However, if $W \subset \overline{L}_{D,k}$ (because $\overline{L}_{Y,k}=\emptyset$), then (17) is violated, which is a contradiction. If *W* is unmeasured, then either (15) is violated or (17) is violated, which is a contradiction. $\square$

### Lemma 6

If the dismissible component conditions hold when we define $L_{D,K}=\emptyset$, then $A_D$ partial isolation holds.

### Proof

The proof is analogous to the proof of lemma 5.

### Lemma 7

If the dismissible component conditions hold for both the partition $L_{D,k}=\emptyset , L_{Y,k}=L_k$ and the partition $L_{Y,k}=L_k, L_{D,k}=\emptyset$, then full isolation holds.

### Proof

It follows immediately from Lemma 5 and Lemma 6, because full isolation holds by definition if both $A_Y$ partial isolation and $A_D$ partial isolation holds. $\square$

### Lemma 8

If the dismissible component conditions hold for both the partition $L_{D,k}=\emptyset , L_{Y,k}=L_k$ and the partition $L_{Y,k}=L_k, L_{D,k}=\emptyset$, then

### Proof

We give a proof by contradiction. Suppose that the dismissible component conditions hold for both the partition $L_{D,k}=\emptyset , L_{Y,k}=L_k$ and the partition $L_{Y,k}=L_k, L_{D,k}=\emptyset$, and there is a conditional dependence such that

$$\begin{aligned} L_{k} \not \!\perp \!\!\!\perp A \mid D_{k}=Y_{k}=0, \bar{L}_{k-1}, \end{aligned}$$

for at least one $k=0, \ldots , K$. Using the rules of d-separation (Pearl 2009), we will consider the 4 possible ways in which *A* and $L_k$ can be d-connected, conditional on $D_{k}=Y_{k}=0, \bar{L}_{k-1}$.

Suppose that the conditional dependence is due to a direct arrow from *A* into $L_k$. Then, under the generalized decomposition assumption, either there is a direct arrow from $A_D$ into $L_k$ or a direct arrow from $A_Y$ into $L_k$, and these arrows would, repsectively, violate (16) under the partition $L_{Y,k}=L_k, L_{D,k}=\emptyset$ and (17) under the partition $L_{D,k}=L_k, L_{Y,k}=\emptyset$, which is a contradiction.

Suppose that the conditional dependence is due the unmeasured common cause *W* of of *A* and $L_k$. Then, under the generalized decomposition assumption, either (i) *W* is a common cause of either $A_Y$ and $L_k$ or (ii) *W* is a common cause of $A_D$ and $L_k$. However, (i) or (ii) would violate dismissible component condition (16) or (17), which is a contradiction.

Suppose that the conditional dependence is due to an unblocked path due to conditioning on $D_{k}=0, Y_{k}=0$ and $\bar{L}_{k-1}$, that is, by conditioning on a collider or a descendant of a collider. Then, under the generalized decomposition assumption, this path would lead to a conditional dependence between either $L_k$ and $A_Y$ or $L_k$ and $A_D$. Any such path would violate (16) or (17), which is a contradiction of the result in Lemma 7.

Finally, suppose that the conditional dependence is due to a direct arrow from $L_k$ where $k =1,\ldots , K$ into *A*. This would violate our assumption of a temporal order, that is, it would imply that *A* occurs after $L_k$, which is a contradiction. $\square$

## D Proof of weighted representation of (20)

First, using laws of probability we can re-formulate the weights $$W_{L_{Y},k} $$ and $$W_{L_{D},k}$$,

$$\begin{aligned} W_{L_{D},k}&= \frac{\prod _{j=0}^{k} \Pr (A = a_D \mid C_{j}= Y_{j} = D_{j} = 0, L_{D,j} = l_{D,j}, \bar{L}_{j-1} = \bar{l}_{j-1})}{\prod _{j=0}^{k} \Pr (A = a_Y \mid C_{j}= Y_{j} = D_{j} = 0, L_{D,j} = l_{D,j}, \bar{L}_{j-1} = \bar{l}_{j-1})} \\&\quad \times \frac{\prod _{j=0}^{k} \Pr (A = a_Y \mid C_{j}= Y_{j} = D_{j} = 0, \bar{L}_{j-1} = \bar{l}_{j-1})}{\prod _{j=0}^{k} \Pr (A = a_D \mid C_{j}= Y_{j} = D_{j} = 0, \bar{L}_{j-1} = \bar{l}_{j-1})} \\&= \frac{ \prod _{j=0}^{k} \frac{\Pr (A = a_D \mid C_{j}= Y_{j} = D_{j} = 0, L_{D,j} = l_{D,j}, \bar{L}_{j-1} = \bar{l}_{j-1}) \Pr ( C_{j}= Y_{j} = D_{j} = 0, L_{D,j} = l_{D,j}, \bar{L}_{j-1} = \bar{l}_{j-1}) }{ \Pr (C_{j}= Y_{j} = D_{j} = 0,\bar{L}_{j-1} = \bar{l}_{j-1}, A = a_D) } }{ \prod _{j=0}^{k} \frac{\Pr (A = a_Y \mid C_{j}= Y_{j} = D_{j} = 0, L_{D,j} = l_{D,j}, \bar{L}_{j-1} = \bar{l}_{j-1}) f( C_{j}= Y_{j} = D_{j} = 0, L_{D,j} = l_{D,j}, \bar{L}_{j-1} = \bar{l}_{j-1}) }{ \Pr (C_{j}= Y_{j} = D_{j} = 0,\bar{L}_{j-1} = \bar{l}_{j-1}, A = a_Y) } } \\&= \frac{\prod _{j=0}^{k} f(L_{D,j} = l_{D,j} \mid C_{j}= Y_{j} = D_{j} = 0, \bar{L}_{j-1} = \bar{l}_{j-1}, A = a_D) }{ \prod _{j=0}^{k} f(L_{D,j} = l_{D,j} \mid C_{j}= Y_{j} = D_{j} = 0, \bar{L}_{j-1} = \bar{l}_{j-1}, A = a_Y) }, \end{aligned}$$

and

$$\begin{aligned} W_{L_{Y},k}&= \frac{\prod _{j=0}^{k} \Pr (A = a_Y \mid C_{j}= Y_{j} = D_{j} = 0, \bar{L}_{j} = \bar{l}_{j})}{\prod _{j=0}^{k}\Pr (A = a_D \mid C_{j}= Y_{j} = D_{j} = 0, \bar{L}_{j} = \bar{l}_{j})} \\&\quad \times \frac{\prod _{j=0}^{k} \Pr (A = a_D \mid C_{j}= Y_{j} = D_{j} = 0,L_{D,j} = l_{D,j}, \bar{L}_{j-1} = \bar{l}_{j-1})}{\prod _{j=0}^{k} \Pr (A = a_Y \mid C_{j}= Y_{j} = D_{j} = 0,L_{D,j} = l_{D,j}, \bar{L}_{j-1} = \bar{l}_{j-1})} \\&= \frac{ \prod _{j=0}^{k} \frac{\Pr (A = a_Y \mid C_{j}= Y_{j} = D_{j} = 0, \bar{L}_{j} = \bar{l}_{j}) \Pr ( C_{j}= Y_{j} = D_{j} = 0, \bar{L}_{j} = \bar{l}_{j}) }{ f(C_{j}= Y_{j} = D_{j} = 0,L_{D,j} = l_{D,j}, \bar{L}_{j-1} = \bar{l}_{j-1}, A = a_Y) } }{ \prod _{j=0}^{k} \frac{\Pr (A = a_D \mid C_{j}= Y_{j} = D_{j} = 0, \bar{L}_{j} = \bar{l}_{j}) f( C_{j}= Y_{j} = D_{j} = 0, \bar{L}_{j} = \bar{l}_{j}) }{ f(C_{j}= Y_{j} = D_{j} = 0,L_{D,j} = l_{D,j}, \bar{L}_{j-1} = \bar{l}_{j-1}, A = a_D) } } \\&= \frac{\prod _{j=0}^{k} f(L_{Y,j} {=} l_{Y,j} \mid C_{j}{=} Y_{j} {=} D_{j} {=} 0,L_{D,j} {=} l_{D,j}, \bar{L}_{j-1} {=} \bar{l}_{j-1}, A {=} a_Y) }{ \prod _{j=0}^{k} f(L_{Y,j} {=} l_{Y,j} \mid C_{j}{=} Y_{j} {=} D_{j} {=} 0,L_{D,j} {=} l_{D,j}, \bar{L}_{j-1} {=} \bar{l}_{j-1}, A {=} a_D) }. \end{aligned}$$

Define

$$\begin{aligned} W'_{C,k} (a_Y) = \frac{1 }{ \prod _{j=0}^{k} \Pr (C_{j+1}=0 \mid C_{j}=D_{j}= Y_{j}=0, \bar{L}_{j} = \bar{l}_{j}, A = a_Y) }. \end{aligned}$$

Consider the expression

$$\begin{aligned} E&[ W_{C,k}(a_Y) W_{D,k} W_{L_{D},k} Y_{k+1} (1-Y_{k}) (1-D_{k+1}) \mid A=a_Y] \\&\quad = E [ W'_{C,k} (a_Y) W_{D,k} W_{L_{D},k} Y_{k+1} (1-Y_{k}) (1-D_{k+1}) (1-C_{k+1}) \mid A=a_Y] \\&\quad = \sum _{\bar{l}_k} \sum _{\bar{y}_{k+1}} \sum _{\bar{d}_{k+1}} [ f(\bar{y}_{k+1}, d_{k+1},c_{k+1},\bar{l}_k \mid A = a_Y) W'_{C,k} (a) W_{D,k} W_{L_{D},k} \\&\quad \quad \times y_{k+1} (1-y_{k}) (1-d_{k+1}) (1-c_{k+1}) ] \\&\quad = \sum _{\bar{l}_k} [\Pr (Y_{k+1}=1,Y_k=D_{k+1}=C_{k+1}=0,\overline{L}_k = \overline{l}_k \mid A = a_Y) W'_{C,k} (a_Y) W_{D,k} W_{L_{D},k}] \\&\quad = \sum _{\bar{l}_k} [ \Pr (Y_{k+1}=1 \mid Y_k= D_{k+1}=C_{k+1}=0,\overline{L}_k = \overline{l}_k, A=a_Y)\\&\quad \Pr ( D_{k+1}=0 \mid \bar{C}_{k+1}= \bar{D}_k= \bar{Y}_k =0,\overline{L}_k = \overline{l}_k,A=a_Y ) \\&\quad \quad \times \Pr (C_{k+1}= 0 \mid \bar{D}_k= \bar{Y}_{k}= \bar{C}_{k} =0,\overline{L}_k = \overline{l}_k, A=a_Y) f( \bar{l}_{k} \mid \bar{C}_{k}= \bar{D}_k= \bar{Y}_{k} =0, A=a_Y) \\&\quad \quad \times \Pr (\bar{Y}_{k}=\bar{D}_{k}=\bar{C}_{k}=0 \mid A= a_Y ) \\&\quad \quad \times W'_{C,k} (a_Y) W_{D,k} W_{L_{D},k}] \\&\quad = \sum _{\bar{l}_k} [ \Pr (Y_{k+1}=1 \mid Y_k= D_{k+1}=C_{k+1}=0,\overline{L}_k = \overline{l}_k,A=a_Y )\\&\quad \Pr ( D_{k+1}=0 \mid \bar{C}_{k+1}= \bar{D}_k= \bar{Y}_k =0,\overline{L}_k = \overline{l}_k,A=a_Y ) \\&\quad \times \Pr (C_{k+1}= 0 \mid \bar{D}_k= \bar{Y}_{k}= \bar{C}_{k} =0,\overline{L}_k = \overline{l}_k, A= a_Y) \\&\quad \times f(l_{k} \mid \bar{Y}_{k}=\bar{D}_{k}=\bar{C}_{k}= 0,\overline{L}_{k-1} = \overline{l}_{k-1}, A= a_Y) \\&\quad \quad \times \Pr (\bar{Y}_{k}=\bar{D}_{k}=\bar{C}_{k}=0,\bar{L}_{k-1}={l}_{k-1} \mid A= a_Y) \\&\quad \quad \times W'_{C,k} (a_Y) W_{D,k} W_{L_{D},k}], \end{aligned}$$

where we use the definition of expected value in the second equation, the fact that $Y_k$ and $D_k$ are binary in the third equation, laws of probability in the fourth and fifth equation.

We use laws of probability to express $f(\bar{Y}_{k}=\bar{D}_{k}=\bar{C}_{k}=0, \bar{l}_{k-1} \mid A= a_Y )$ as

$$\begin{aligned}&\Pr (Y_{k}=0 \mid C_{k}=D_{k}= Y_{k-1}=0, \overline{L}_{k-1} = \overline{l}_{k-1}, A = a_Y) \\&\quad \times \Pr (D_{k}=0 \mid C_{k}=D_{k-1}= Y_{k-1}=0, \overline{L}_{k-1} = \overline{l}_{k-1}, A = a_Y) \\&\quad \times \Pr (C_{k}= 0 \mid D_{k-1} = Y_{k-1}= C_{k-1} =0,\overline{L}_{k-2} = \overline{l}_{k-2}, A = a_Y) \\&\quad \times f(l_{k-1} \mid C_{k-1}=D_{k-1}= Y_{k-1}=0,\overline{L}_{k-2} = \overline{l}_{k-2}, A = a_Y) \\&\quad \times f(\bar{Y}_{k-1}=\bar{D}_{k-1}=0, \overline{L}_{k-2} = \overline{l}_{k-2}, \bar{C}_{k-1}=0 \mid A= a_Y ), \end{aligned}$$

where any variable indexed with a number $m < 0$ is defined to be the empty set.

Arguing iteratively for $k-1,k-2,...,0$ we find that

$$\begin{aligned} E [ W'_{C,k}&(a_Y) W_{D,k} W_{L_{D},k} Y_{k+1} (1-Y_{k}) (1-D_{k+1}) (1- C_{k+1}) \mid A=a_Y ] \\ = \sum _{\bar{l_k}}&\Big [ \Pr (Y_{k+1}=1 \mid Y_k= D_{k+1}=C_{k+1}=0,\overline{L}_k = \overline{l}_k, A=a_Y) \\ \prod _{j=0}^{k}&\big \{ \Pr (D_{j+1}=0 \mid C_{j+1}=D_{j}= Y_{j}=0, \bar{L}_{j} = \bar{l}_{j}, A = a_Y) \\&\quad \times \Pr (Y_{j}=0 \mid C_{j}=D_{j}= Y_{j-1}=0, \bar{L}_{j-1} = \bar{l}_{j-1}, A = a_Y) \\&\quad \times \Pr (C_{j+1}= 0 \mid \bar{D}_j= \bar{Y}_{j}= \bar{C}_{j} =0,\bar{L}_{j} = \bar{l}_{j}, A=a_Y) \\&\quad \times f(L_{j}=l_{j} \mid C_{j}=D_{j}= Y_{j}=0,\bar{L}_{j-1} = \bar{l}_{j-1}, A = a_Y) \big \} \\&\quad \times W'_{C,k} (a_Y) W_{D,k} W_{L_{D},k} \Big ] \\ = \sum _{\bar{l_k}}&\Big [ \Pr (Y_{k+1}=1 \mid Y_k= D_{k+1}=C_{k+1}=0,\overline{L}_k = \overline{l}_k, A=a_Y) \\ \prod _{j=0}^{k}&\big \{ \Pr (D_{j+1}=0 \mid C_{j+1}=D_{j}= Y_{j}=0, \bar{L}_{j} = \bar{l}_{j}, A = a_Y) \\&\quad \times \Pr (Y_{j}=0 \mid C_{j}=D_{j}= Y_{j-1}=0, \bar{L}_{j-1} = \bar{l}_{j-1}, A = a_Y) \\&\quad \times \Pr (C_{j+1}= 0 \mid \bar{D}_j= \bar{Y}_{j}= \bar{C}_{j} =0,\bar{L}_{j} = \bar{l}_{j}, A = a_Y) \\&\quad \times f(L_{Y,j} = l_{Y,j} \mid C_{j-1}= Y_{j-1} = D_{j} = 0, \bar{L}_{j-1} = \bar{l}_{j-1},\\&\quad L_{D,j} = l_{D,j}, A = a_Y) \\&f(L_{D,j} = l_{D,j} \mid C_{j}= Y_{j} = D_{j} = 0, \bar{L}_{j-1} = \bar{l}_{j-1}, A = a_Y) \big \} \\&\quad \times W'_{C,k} (a_Y) W_{D,k} W_{L_{D},k} \Big ], \end{aligned}$$

where we use that $L_k = (L_{Y,k},L_{D,k})$ in the second equality.

By plugging in the expression for $W'_{C,k} (a_Y)$, we get

$$\begin{aligned}&= \sum _{\bar{l_k}} [ \Pr (Y_{k+1}=1 \mid Y_k= D_{k+1}=C_{k+1}=0,\bar{L}_k=\bar{l}_k, A=a_Y) \\&\quad \times \prod _{j=0}^{k} \big \{ \Pr (D_{j+1}=0 \mid C_{j+1}=D_{j}= Y_{j}=0, \bar{L}_{j} = \bar{l}_{j}, A = a_Y) \\&\quad \times \Pr (Y_{j}=0 \mid C_{j}=D_{j}= Y_{j-1}=0, \bar{L}_{j-1} = \bar{l}_{j-1}, A = a_Y) \\&\quad \times f(L_{Y,j} = l_{Y,j} \mid C_{j}= Y_{j} = D_{j} = 0, \bar{L}_{j-1} = \bar{l}_{j-1}, L_{D,j} = l_{D,j}, A = a_Y) \\&\quad \times f(L_{D,j} = l_{D,j} \mid C_{j}= Y_{j} = D_{j} = 0, \bar{L}_{j-1} = \bar{l}_{j-1}, A = a_Y) \big \} \\&\quad \times W_{D,k} W_{L_{D},k}], \\ \end{aligned}$$

By plugging in the expression for the weights $W_{L_{D},k}$ and $W_{D,k}$ we obtain

$$\begin{aligned}&= \sum _{\bar{l_k}} [ \Pr (Y_{k+1}=1 \mid Y_k= D_{k+1}=C_{k+1}=0,\bar{L}_k=\bar{l}_k, A=a_Y) \\&\quad \times \prod _{j=0}^{k} \big \{ \Pr (D_{j+1}=0 \mid C_{j+1}=D_{j}= Y_{j}=0, \bar{L}_{j} = \bar{l}_{j}, A = a_D) \\&\quad \times \Pr (Y_{j}=0 \mid C_{j}=D_{j}= Y_{j-1}=0, \bar{L}_{j-1} = \bar{l}_{j-1}, A = a_Y) \\&\quad \times f(L_{Y,j} = l_{Y,j} \mid C_{j}= Y_{j} = D_{j} = 0, \bar{L}_{j-1} = \bar{l}_{j-1}, L_{D,j} = l_{D,j}, A = a_Y) \\&\quad \times f(L_{D,j} = l_{D,j} \mid C_{j}= Y_{j} = D_{j} = 0, \bar{L}_{j-1} = \bar{l}_{j-1}, A = a_D) \big \}, \end{aligned}$$

and the final expression is equal to (20).

## E Estimation algorithms

Here we describe an algorithm to estimate the separable effects using estimators based on (21); i.e. the estimator $\hat{\nu }_{1,k}$ described in Sect. 7. We initially construct our input data set such that each subject has $K^*+1$ lines, indexed by $k = 0,\dots ,K^*$, and there are measurements of $(A, C_{k+1},D_{k+1}, Y_{k+1},\bar{L}_{k+1})$ on each line *k*. For each subject, $K^*=K$ if $C_{K+1}=D_{K+1}=Y_{K+1}=0$, otherwise $K^*=m$, where $C_{m}=D_{m}=Y_{m}=0$ and either $C_{m+1}=1$, $D_{m+1}=1$ or $Y_{m+1}=1$. Due to the temporal ordering, we do the following: if $C_{k+1}=1$, then $D_{k+1}$ and $Y_{k+1}$ are set missing. Similarly, if $C_{k+1}=0$ and $D_{k+1}=1$, then $Y_{k+1}=1$ is set missing. Then we do the following to estimate (21) at *K*:

1. Using all subject-intervals records, i.e. all lines in the data set, obtain $\hat{\beta }_D$ by fitting a parametric model (e.g. pooled logistic regression model) with dependent variable $D_{k+1}$ and independent variables a specified function of $k = 0, \dots K$, $\bar{L}_k$ and *A*.
2. Using all subject-intervals records, obtain $\hat{\beta }_C$ by fitting a parametric model (e.g. pooled logistic regression model) with dependent variable $C_{k+1}$ and independent variables a specified function of $k = 0, \dots K$, $\bar{L}_k$ and *A*.
3. Using all subject-intervals records, estimate $\hat{\beta }_{L1}$ by fitting a parametric model with dependent variable *A* and independent variables a specified function of $k = 0, \dots K$, $\bar{L}_k$ and *A*.
4. Using all subject-intervals records, estimate $\hat{\beta }_{L2}$ by fitting a parametric model with dependent variable *A* and independent variables a specified function of $k = 0, \dots K$, $\bar{L}_{k-1}$,$L_{D,k}$ and *A*, ensuring that the models used to fit $\hat{\beta }_{L1}$ and $\hat{\beta }_{L2}$ are compatible. Notice that this step is redundant if we can define a $L_k$ partition such that $L_{D,k} = \emptyset , k = 0, \dots K$, which implies that $A_D$ partial isolation holds.
5. For subject *i*, attach a weight to line *k* with predicted outcome probabilities derived from the parametric models indexed by parameters $\hat{\beta }_{D}, \hat{\beta }_{L1},\hat{\beta }_{L2}$ and $\hat{\beta }_{C}$ to estimate $\hat{W}_{1,i,k}( \hat{\beta }_1) = \hat{W}_{i,D,k} (\hat{\beta }_{D}) \hat{W}_{i,L_{D},k} (\hat{\beta }_{L1},\hat{\beta }_{L2}) \hat{W}_{i,C,k} (\hat{\beta }_{C})$.
6. Compute an estimate of $\Pr (Y^{a_Y,a_D,\bar{c}=0}_{K+1})$ from
  $$\begin{aligned} \frac{1}{\sum ^{n}_{j=1}I(A_j=a_Y)} \sum ^{n}_{i=1} \sum _{k=0}^{K} \hat{W}_{1,i,k}(\hat{\beta }_1) Y_{i,k+1} (1-Y_{i,k}) (1-D_{i,k+1})I(A_i=a_Y). \end{aligned}$$

An estimator based on (22) could be derived analogously, where step (1) we would fit a model with $Y_{k+1}$ as dependent variable, in step (4) we would fit a model where we replace $L_{D,k}$ with $L_{Y,k}$, and we finally compute an estimate of $\Pr (Y^{a_Y,a_D,\bar{c}=0}_{K+1})$ from

$$\begin{aligned} \frac{1}{\sum ^{n}_{j=1}I(A_j=a_D)} \sum ^{n}_{i=1} \sum _{k=0}^{K} \hat{W}_{2,i,k}(\hat{\beta }_1) Y_{i,k+1} (1-Y_{i,k}) (1-D_{i,k+1})I(A_i=a_D). \end{aligned}$$

## F Proof of sensitivity analysis

This section includes a proof of the estimating equation for the sensitivity analysis in Sect. 7.3.

### Proof

The following equality holds by definition of $t_{k}(\bar{l}_k, a_Y)$,

54 $$\begin{aligned}&\Pr (Y^{a_Y,a_D=0,\bar{c}= 0}_{k+1} = 1 \mid Y^{a_Y,a_D=0,\bar{c}= 0}_k = D^{a_Y,a_D=0,\bar{c}= 0}_{k+1} = 0,\bar{L}^{a_Y,a_D=0,\bar{c}= 0}_{k} = \bar{l}_{k}) \\&\quad = t_{k}(\bar{l}_k, a_Y)+\Pr (Y^{a_Y,a_D=1,\bar{c}= 0}_{k+1} = 1 \mid Y^{a_Y,a_D=1,\bar{c}= 0}_k = D^{a_Y,a_D=1,\bar{c}= 0}_{k+1} = 0,\nonumber \\&\quad \bar{L}^{a_Y,a_D=1,\bar{c}= 0}_{k} = \bar{l}_{k}). \end{aligned}$$

While (38) of Lemma 1 is violated in our setting where dismissible component condition (14) is violated, note that (39)–(41) of Lemma 1 holds and that Lemma 2 holds regardless of violations of the dismissible component conditions. Thus, following analogous steps as in the proof of Theorem 1, we use (28)–(33), (35)–(37), as well as (54) instead of (34), to obtain the following identification formula for settings where $a_Y \ne a_D$,

$$\begin{aligned} \Pr (&Y_{k+1}^{a_Y,a_D,\bar{c}=0}=1) \\&= \sum _{\bar{l}_K} \Big [ \sum _{s=0}^{K} (-1)^{a_D} t_{k}(\bar{l}_k, a_Y) + \Pr (Y_{s+1}=1 \mid C_{s+1}= D_{s+1}= Y_{s}=0,\nonumber \\&\quad \bar{L}_{s} = \bar{l}_{s}, A = a_Y) \\&\prod _{j=0}^{s} \big \{ \Pr (D_{j+1}=0 \mid C_{j+1}=D_{j}= Y_{j}=0, \bar{L}_{j} = \bar{l}_{j}, A = a_D) \\&\quad \times [ (-1)^{a_D} t_{k}(\bar{l}_k, a_Y) + \Pr (Y_{j}=0 \mid C_{j}=D_{j}= Y_{j-1}=0,\nonumber \\&\quad \bar{L}_{j-1} = \bar{l}_{j-1}, A = a_Y)] \\&\quad \times f(L_{Y,j} = l_{Y,j} \mid C_{j}= Y_{j} = D_{j} = 0, \bar{L}_{j-1} = \bar{l}_{j-1}, L_{D,j} = l_{D,j}, A = a_Y) \\&\quad \times f(L_{D,j} = l_{D,j} \mid C_{j}= Y_{j} = D_{j} = 0, \bar{L}_{j-1} = \bar{l}_{j-1}, A = a_D) \big \} \Big ], \end{aligned}$$

and a weighted representation of this identification formula is analogous to identification formula (22), where $\hat{W}_{2,s}$ is replaced by $W^{\dagger }_{Y,s}$, which can be shown by an argument that is analogous to the proof in Appendix D. $\square$

## Footnotes

1 Many authors use the term “censoring by death” without reference to an estimand. This terminology is appropriate when the estimand is defined with respect to interventions that eliminate competing events, but it is misleading in general. See Young et al. (2020) for details. Here we intentionally avoid the term “censoring by death” because we do not consider such estimands.

2 The extended graph in Fig. 4a is the generalization of Richardson and Robins’s extended graph in their Figure 6A and the graph in Fig. 4b is the generalization of Richardson and Robins’s graph in their Figure 6B to settings with time dependent confounders, mediators and outcomes.

## References

[CR1] Aalen O, Johansen S (1978) An empirical transition matrix for non-homogeneous Markov chains based on censored observations. Scand J Stat 141–150

[CR2] Aalen O, Stensrud MJ, Didelez V, Daniel R, Røysland K, Strohmaier S (2019) Time-dependent mediators in survival analysis: modeling direct and indirect effects with the additive hazards model. Biom J 10.1002/bimj.201800263 30779372

[CR3] Avin C, Shpitser I, Pearl J (2005) Identifiability of path-specific effects

[CR4] Chernozhukov V, Chetverikov D, Demirer M, Duflo E, Hansen C, Newey W, Robins J (2018) Double/debiased machine learning for treatment and structural parameters. Econom J 21(1):C1–C68, 01

[CR5] Cui Y, Tchetgen Tchetgen EJ (2019) Bias-aware model selection for machine learning of doubly robust functionals. arXiv preprint arXiv:1911.02029

[CR6] Didelez V (2018) Defining causal mediation with a longitudinal mediator and a survival outcome. Lifetime Data Anal 1–18 10.1007/s10985-018-9449-0 30218418

[CR7] Hernán MA (2010). The hazards of hazard ratios. Epidemiology (Cambridge, MA).

[CR8] Hernan MA, Robins JM (2018) Causal inference. CRC Boca Raton, FL

[CR9] Martinussen T, Vansteelandt S, Andersen PK (2020). Subtleties in the interpretation of hazard contrasts. Lifetime Data Anal.

[CR10] Pearl J (2009). Causality.

[CR11] Richardson TS, Robins JM (2013) Single world intervention graphs (swigs): a unification of the counterfactual and graphical approaches to causality. Center for the Statistics and the Social Sciences , University of Washington Series, Working Paper 128(30):2013

[CR12] Ridker PM, Pradhan A, MacFadyen JG, Libby P, Glynn RJ (2012). Cardiovascular benefits and diabetes risks of statin therapy in primary prevention: an analysis from the jupiter trial. The Lancet.

[CR13] Robins JM (1986). A new approach to causal inference in mortality studies with a sustained exposure period—application to control of the healthy worker survivor effect. Math Model.

[CR14] Robins JM (2016) Direct and indirect effects. Presentation at the UK causal inference conference in London

[CR15] Robins JM, Greenland S (1992) Identifiability and exchangeability for direct and indirect effects. Epidemiology 143–155 10.1097/00001648-199203000-00013 1576220

[CR16] Robins JM, Richardson TS (2010) Alternative graphical causal models and the identification of direct effects, pp 103–158

[CR17] Robins JM, Rotnitzky A, Zhao LP (1994). Estimation of regression coefficients when some regressors are not always observed. J Am Stat Assoc.

[CR18] Robins JM, Li L, Rajarshi M, Eric T, van der Vaart A (2017). Minimax estimation of a functional on a structured high-dimensional model. Ann Stat.

[CR19] Robins JM, Richardson TS, Shpitser I (2020) An interventionist approach to mediation analysis. arXiv preprint arXiv:2008.06019

[CR20] Sattar N, Preiss D, Murray HM, Welsh P, Buckley BM, de Craen AJM, Seshasai SRK, McMurray JJ, Freeman DJ, Wouter Jukema J (2010). Statins and risk of incident diabetes: a collaborative meta-analysis of randomised statin trials. The Lancet.

[CR21] Shpitser I (2013). Counterfactual graphical models for longitudinal mediation analysis with unobserved confounding. Cogn Sci.

[CR22] Shpitser I, Richardson TS, Robins JM (2020) Multivariate counterfactual systems and causal graphical models. arXiv preprint arXiv:2008.06017

[CR23] SPRINT Research Group (2015). A randomized trial of intensive versus standard blood-pressure control. N Engl J Med.

[CR24] Stensrud MJ, Hernán MA (2020). Why test for proportional hazards?. Jama.

[CR25] Stensrud MJ, Young JG, Didelez V, Robins JM, Hernán MA (2020) Separable effects for causal inference in the presence of competing events. J Am Stat Assoc 1–23

[CR26] Tchetgen Tchetgen EJ (2013). Inverse odds ratio-weighted estimation for causal mediation analysis. Stat Med.

[CR27] Tchetgen Tchetgen EJ (2014). Identification and estimation of survivor average causal effects. Stat Med.

[CR28] Turo R, Smolski M, Esler R, Kujawa ML, Bromage SJ, Oakley N, Adeyoju A, Brown SCW, Brough R, Sinclair A (2014). Diethylstilboestrol for the treatment of prostate cancer: past, present and future. Scand J Urol.

[CR29] Van der Laan MJ, Rose S (2018). Targeted learning in data science.

[CR30] Verma T, Pearl J (1991) Equivalence and synthesis of causal models. UCLA, Computer Science Department

[CR31] Williamson JD, Supiano MA, Applegate WB, Berlowitz DR, Campbell RC, Chertow GM, Fine LJ, Haley WE, Hawfield AT, Ix JH (2016). Intensive vs standard blood pressure control and cardiovascular disease outcomes in adults aged $$le $$ 75 years: a randomized clinical trial. Jama.

[CR32] Young JG, Stensrud MJ, Tchetgen Tchetgen EJ, Hernán MA (2020) A causal framework for classical statistical estimands in failure-time settings with competing events. Stat Med 39(8):1199–1236 10.1002/sim.8471 PMC7811594 31985089

