# Supplementary material for: A generalized theory of separable effects in competing event settings
Source: Lifetime Data Anal. 2021 Sep 1;27(4):588–631. doi: 10.1007/s10985-021-09530-8 (PMC8536652; doi:10.1007/s10985-021-09530-8)
Supplement: Supplementary file 2 — Supplementary material 2 (pdf 194 KB) [file 10985_2021_9530_MOESM2_ESM.pdf]

Noname manuscript No.
(will be inserted by the editor)

Supplementary material: A generalized theory of separable effects in competing event settings

Anonymous

the date of receipt and acceptance should be inserted later

Key words: Causal inference; Competing events; Effect decomposition; G-formula; Hazard functions; Separable effects.

Address(es) of author(s) should be given

1 Treatment decomposition of A into A_Y, A_D and A_Z

Hitherto we have described settings in which the treatment is decomposed into 2 components, A_D and A_Y. Consider now a hypothetical treatment decomposition into 3 components A_D, A_Y and A_Z, as illustrated in Figure 1, which is similar to Robins and Richardson's decomposition in a mediation setting [1, Figure 6(d)]. Analogous to the 2 way decomposition, we define a generalized decomposition assumption:

3 way generalized decomposition assumption: The treatment A can be decomposed into three binary components $A_Y \in \{0,1\}$, $A_D \in \{0,1\}$ and $A_Z \in \{0,1\}$ such that, in the observed data, the following determinism holds

$$A \equiv A_D \equiv A_Y \equiv A_Z, \quad (1)$$

but, in a future study, A_Y,A_D and A_Z could be assigned different values under a hypothetical intervention. For any individual in the study population and for $k \in \{0,\dots,K\}$, let $Y_{k+1}^{a_Y,a_D,a_Z}$ be the indicator of the event of interest by interval $k+1$ had, possibly contrary to fact, he/she been assigned to $A_Y = a_Y$, $A_D = a_D$ and $A_Z = a_Z$, where $a_Y, a_D, a_Z \in \{0,1\}$. We assume that an intervention that assigns $A = a$ results in the same outcome as an intervention that assigns $A_Y = A_D = A_Z = a$, that is,

$$Q_{k+1}^{a_Y=a_D=a_Z=a} = Q_{k+1}^{a}, \quad (2)$$

for $Q_{k+1} \in \{Y_{k+1}, D_{k+1}, Z_{k+1}\}$. Analogous to the 2 way decomposition, the 3 way decomposition may be practically interesting in settings where we can conceive interventions on all 3 components of A. Furthermore, in settings where Z_k partition fails, it may be possible to define a 3 way decomposition that allows identifiability of separable effects. For example, Figure 1 can represent an alternative decomposition of the setting described in Figure 5a, where Z_k partition fails.

To define identifiability conditions that apply to settings with 3 way decompositions, we continue to use superscripts to denote counterfactuals and for notational simplicity we consider settings without censoring, such that e.g. $Y_{k+1}^{a_Y,a_D,a_Z}$ is the counterfactual value of Y_{k+1} if, possibly contrary to fact, $A_Y = a_Y, A_D = a_D, A_Z = a_Z \in \{0,1\}$.

Here we will only consider settings that satisfy the following assumptions:

the only causal paths from A_Y to D_{k+1} and $Z_{k+1}, k \in \{0,\dots,K\}$ are through Y_j, $j = 0,...,k$, (3)

the only causal paths from A_D to Y_{k+1} and $Z_{k+1}, k = 0,\dots,K$ are through D_{j+1}, $j = 0,\dots,k$. (4)

the only causal paths from A_Z to Y_{k+1} and $D_{k+1}, k = 0,\dots,K$ are through Z_{j+1}, $j = 0,\dots,k$. (5)

For $k = 0, \ldots, K$, consider the separable effects

$$\Pr(Y_{k+1}^{a_Y=1,a_D,a_Z} = 1) \text{ vs. } \Pr(Y_{k+1}^{a_Y=0,a_D,a_Z} = 1), \tag{6}$$

for $a_D, a_Z \in \{0,1\}$,

$$\Pr(Y_{k+1}^{a_Y,a_D=1,a_Z} = 1) \text{ vs. } \Pr(Y_{k+1}^{a_Y,a_D=0,a_Z} = 1), \tag{7}$$

for $a_Y, a_Z \in \{0,1\}$, and

$$\Pr(Y_{k+1}^{a_Y,a_D,a_Z=1} = 1) \text{ vs. } \Pr(Y_{k+1}^{a_Y,a_D,a_Z=0} = 1), \tag{8}$$

for $a_Y, a_D \in \{0,1\}$.

Similar to the two component decomposition, the total effect can be expressed as a sum of the separable direct and indirect effects, in particular,

$$\begin{aligned}
&\Pr(Y_{k+1}^{a_Y=1,a_D=1,a_Z=1} = 1) - \Pr(Y_{k+1}^{a_Y=0,a_D=1,a_Z=1} = 1) \\
&+ \Pr(Y_{k+1}^{a_Y=0,a_D=1,a_Z=1} = 1) - \Pr(Y_{k+1}^{a_Y=0,a_D=0,a_Z=1} = 1) \\
&+ \Pr(Y_{k+1}^{a_Y=0,a_D=0,a_Z=1} = 1) - \Pr(Y_{k+1}^{a_Y=0,a_D=0,a_Z=0} = 1) \\
&= \Pr(Y_{k+1}^{a=1} = 1) - \Pr(Y_{k+1}^{a=0} = 1).
\end{aligned}$$

1.1 Interpretation of the 3 component decomposition

Under (3)-(5), the 3 way decomposition of A into A_D, A_Y and A_Z allows us to interpret the separable effects as direct and indirect effects; (6) is the effect not emanating from A_D or A_Z, i.e. a separable direct effect, (7) is the separable indirect effect on the event of interest only emanating from A_D, and (8) is the separable indirect effect on the event of interest only emanating from A_Z.

In our running example, where $Z_k = L_k$ encodes the (systolic and diastolic) blood pressure, it is not obvious that the 3 part decomposition is of interest; to interpret effects defined by the 3 part decomposition, we would need to conceptualize a treatment decomposition of blood pressure therapy into 3 components: the A_D component could now be defined as the component that exerts effects on mortality not through blood pressure reduction or kidney injury; that is, the substantive meaning of an intervention on A_D fundamentally changes. The A_Z component would affect the outcome of interest only through blood pressure reduction; the effect exerted by A_Z is analogous to an indirect mediation effect described by Didelez [2] under an agnostic causal model, but in our setting we also allow for competing risks. We note that under this 3 way decomposition, the A_Y component is identical to the A_Y component in the 2 way decomposition, that is, the component of blood pressure therapy only exerting direct effects on kidney injury not through blood pressure reduction.

In other settings, however, the 3 part decomposition may be feasible. For example, Robins and Richardson [1, Figure 6(d)] consider a similar decomposition in a conceptual example on the effect of cigarettes on lung cancer; they consider the effect of cigarettes smoking through nicotine, tar and other pathways.

1.2 Identification of the 3 component decomposition

The identifiability conditions are straightforward extensions of the conditions in Section 6. Now we must identify

$$\Pr(Y_{k+1}^{a_Y,a_D,a_Z} = 1) \text{ for } a_Y, a_D, a_Z \in \{0,1\}.$$

First the exchangeability, consistency and positivity conditions are identical to the condition in Section 6. The dismissible component conditions read

$$Y_{k+1}(G) \perp\!\!\!\perp (A_D(G), A_Z(G)) \mid A_Y(G), Y_k(G) = D_{k+1}(G) = 0, \bar{L}_k(G),$$
$$D_{k+1}(G) \perp\!\!\!\perp (A_Y(G), A_Z(G)) \mid A_D(G), D_k(G) = Y_k(G) = 0, \bar{L}_k(G),$$
$$L_{k+1}(G) \perp\!\!\!\perp (A_Y(G), A_D(G)) \mid A_Z(G), D_{k+1}(G) = Y_{k+1}(G) = 0, \bar{L}_k(G).$$

Under these assumptions we can identify $\Pr(Y_{k+1}^{a_Y,a_D,a_Z} = 1)$ for $k = 0, \ldots, K$ from

$$\sum_{\bar{l}_k} \Big[\sum_{s=0}^{k} \Pr(Y_{s+1} = 1 \mid D_{s+1} = Y_s = 0, \bar{L}_s = \bar{l}_s, A = a_Y)$$
$$\prod_{j=0}^{s} \Big\{ \Pr(D_{j+1} = 0 \mid D_j = Y_j = 0, \bar{L}_j = \bar{l}_j, A = a_D)$$
$$\times \Pr(Y_j = 0 \mid D_j = Y_{j-1} = 0, \bar{L}_{j-1} = \bar{l}_{j-1}, A = a_Y)$$
$$\times f(L_j = l_j \mid Y_j = D_j = 0, \bar{L}_{j-1} = \bar{l}_{j-1}, A = a_Z) \Big\} \Big], \quad (9)$$

which follows from a similar derivation from that in Appendix B. The identifiability conditions under the 3 component decomposition require stronger restrictions on the unmeasured variables, compared to the settings in Section 6; unmeasured common causes of any pair in $(Y_{k+1}, D_{j+1}, L_{m+1}), k, j, m \in \{0, \ldots, K\}$ can violate the dismissible component conditions. In particular, an unmeasured common cause $U_{L,Y}$ of L_k and Y_k will violate the dismissible component condition, as shown in grey in Figure 2.

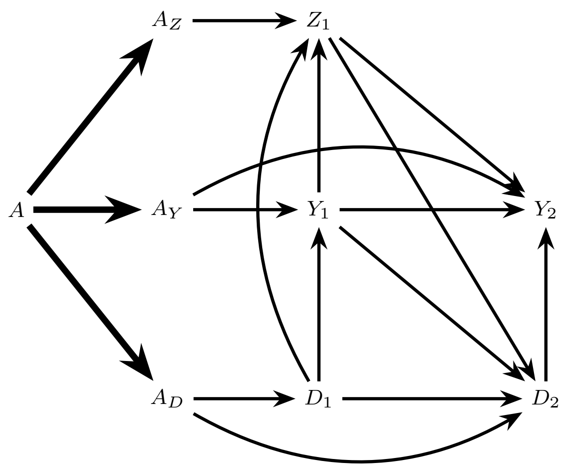

Fig. 1: Treatment A is decomposed into 3 components.

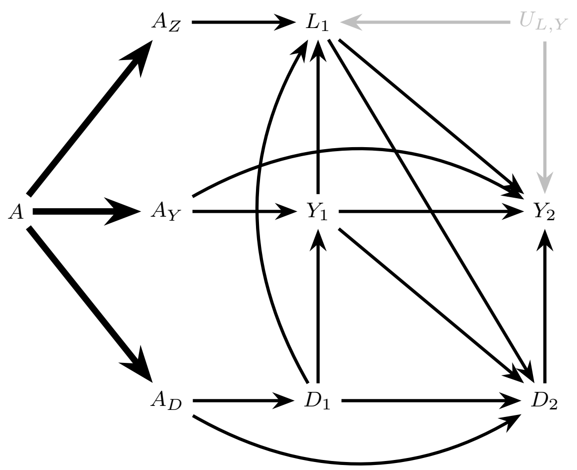

Fig. 2: Treatment is decomposed into 3 components, such that $L_1 = Z_1$. The variable $U_{L,Y}$ would violate the dismissible component conditions here.

References

1. James M Robins and Thomas S Richardson. Alternative graphical causal models and the identification of direct effects. *Causality and psychopathology: Finding the determinants of disorders and their cures*, pages 103–158, 2010.
2. Vanessa Didelez. Defining causal mediation with a longitudinal mediator and a survival outcome. *Lifetime Data Analysis*, pages 1–18, 2018.
